# An experimental and computational study to explore the ion–solvent interactions between selected ionic liquids and dimethylformamide†

**DOI:** 10.1039/d4ra08020c

**Published:** 2025-04-11

**Authors:** Sandeep Kumar, Teshome Abute Lelisho, Indra Bahadur, Thishana Singh

**Affiliations:** a School of Chemistry and Physics, University of KwaZulu-Natal Private Bag X54001 Durban 4000 South Africa singht1@ukzn.ac.za bahadur.indra@nwu.ac.za; b Department of Chemistry, CNCS, Hawassa University Hawassa Ethiopia; c Department of Chemistry, Faculty of Natural and Agricultural Sciences, North-West University (Mafikeng Campus) Private Bag X2046 Mmabatho 2735 South Africa

## Abstract

Solute–solvent, solute–solute and solvent–solvent interactions are examined *via* thermodynamics using apparent molar properties which are temperature dependent and are useful to define the isolated contribution of each component to the non-ideality of the mixture. Apparent molar volumes (*V*_ϕ_) and apparent molar adiabatic compressibilities (*K*_ϕ_) were investigated for three binary mixtures with different anions: 1-butyl-3-methylimidazolium chloride [Bmim][Cl], 1-butyl-1-methylpyrrolidinium chloride [Bmpym][Cl] and 1-butyl-3-methylimidazolium thiocyanide [Bmim][SCN] with dimethylformamide (DMF) at different temperatures (293.15–343.15) K and at ambient pressure. Density (*ρ*) and speed of sound (*u*) of the pure components and their mixtures were recorded. The data was fitted to the Redlich–Mayer polynomial equation to calculate the derived thermodynamic parameters: limiting apparent molar volume (*V*^0^_ϕ_), limiting apparent molar expansion (*E*^0^_ϕ_), thermal expansion coefficients (*α*_p_) and limiting apparent molar adiabatic compressibility (*K*^0^_ϕ_) along with their associated parameters (*S*_v_, *B*_v_, *S*_k_, *B*_k_). The primary focus of this study was to examine the effect of temperature on the anion and cation interaction of the IL with DMF and how these changes affected the IL structure. The computational investigation further examined the IL–solvent interaction energy and described the type of interaction in all three systems.

## Abstract 2

DMF–IL interaction at different temperatures involved calculating various volumetric and thermodynamic properties. DFT calculations with NCI and QTAIM analysis confirmed experimental observations: [Bmim][SCN] system is the most effective complex.

## Graphical abstract

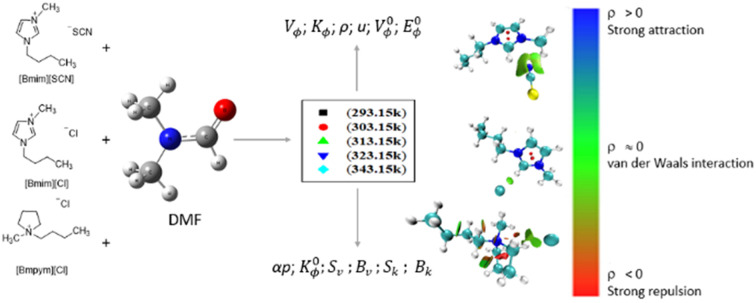

## 1. Introduction

Nowadays, ILs are increasingly used as environmentally acceptable solvents, replacing volatile organic solvents in a variety of industries. This transition has resulted in their extensive use in the chemical synthesis and pharmaceutical sectors, which collectively generate millions of tons of hazardous waste each year. ILs are a type of liquid composed solely of ions and possess unique physical and chemical properties that make them useful in a wide range of applications including solution chemistry, extractions, electrochemistry, separations, and biological processes.^1–5^

The focus of this work was on the [Bmim][Cl], [Bmpym][Cl], and [Bmim][SCN] ILs, which were investigated because they are inexpensive, have good thermal stability, and their ease of use in solubilizing a variety of solutes, including inorganic, organic, and polymeric molecules.^6–9^ [Bmim][Cl] has several potential applications in the pharmaceutical and food industries. One of the main advantages of [Bmim][Cl] is its ability to dissolve a wide range of compounds, including many drug and food ingredients that are insoluble in conventional solvents. It is a promising candidate for enhancing the bioavailability of poorly soluble drugs due to its capacity to solubilize them.

The study of volumetric properties of ILs is especially important. Density, compressibility, and thermal expansion coefficient are examples of the physical characteristics of a substance that are associated with its volumetric features.^10–14^ These characteristics are important in many fields, including material sciences and chemical engineering. However, the primary benefits of ILs are its wide range of tuneable properties.^12–14^ This means it is possible to modify the chemical structure of an IL to tailor its volumetric properties for certain applications. For example, it is possible to create ILs that have very low vapour pressures to be used as solvents in a process which requires high temperatures or pressures.^15–17^ Since ILs are easy to handle and have low melting points they are ideal for the investigation of volumetric properties. Furthermore, they have high polarity and are composed entirely of ions, which can be used to modify the solubility of other substances in the liquid. Overall, the unique properties of ILs make them a valuable tool to examine volumetric properties, with prospective applications ranging from pharmaceuticals to chemical engineering.^18,19^

Dimethylformamide (DMF) is an important polar aprotic solvent that is widely used in various chemical reactions and industrial processes. Its unique properties such as a high boiling point, good solubility in both water and organic solvents, and the ability to form hydrogen bonds with other molecules, make it an ideal solvent for many applications. An important application of DMF is in the determination of excess molar volumes of binary liquid mixtures. The excess molar volumes of these binary liquid mixtures, such as DMF + alcohols, ILs + organic solvents and DMF + ionic liquids were investigated over a range of temperatures, pressures, compositions (concentration), and reported in numerous scientific publications.^20–24^

A thorough knowledge of the structure and properties of ILs is essential to understand the molecular interactions in binary mixtures. Furthermore, to facilitate their application in chemical industrial processes, it is important to understand the thermophysical properties of ILs, since these properties are the foundation for chemical and biological processes. Experimental thermophysical property values enable the development of new predictive relationships and provide useful information about the molecular structure which is of benefit to industry. Hence, the scientific community must have a thorough understanding of the thermophysical properties of liquid mixtures containing ILs. It is evident that the physicochemical characteristics of ILs are significantly influenced by the nature and structure of cations and anions. Variations in the thermophysical properties of ILs, such as density (*ρ*) and speed of sound (*u*) are observed to be highly sensitive to changes in the ion, primarily due to microscopic-level interactions between solvent molecules. Studies on the thermophysical properties of ILs and their mixtures with polar solvents have shown they are suitable for chemical processes. However, to date, no comprehensive and comparative investigation has been done on the properties and structure relationship of imidazolium-based ILs or mixtures of the ammonium-family of ILs with DMF. This includes investigating the effects of variables such as cation size, temperature, composition, and ion–ion interactions.

A few studies have been carried out on the excess molar volumes of DMF mixtures with various compounds, and the findings provide useful information about the molecular interactions between the components in these combinations. In this study, an extensive examination was undertaken to understand the interaction of the carbonyl group in DMF with the polar ions of the ILs. The three ILs used in this investigation were: 1-butyl-3-methylimidazolium thiocyanate [Bmim][SCN], 1-butyl-3-methylimidazolium chloride [Bmim][Cl] and 1-butyl-1-methylpyrrolidinium chloride [Bmpym][Cl]. To the best of our knowledge, these systems have not been reported in literature. There are a few publications that provide a thorough computational analysis in addition to the experimental results on ionic liquids. However, in this study, we have attempted to explain the interactions at an electronic level, through the application of quantum theory of atoms in molecules (QTAIM) and non-covalent interaction (NCI) analysis. The findings are examined in relation to how cations and anions affect the intermolecular interactions between DMF and IL.

## 2. Experimental section

### 2.1 Chemicals

ILs used in this study were purchased from IoLiTec, Germany with a mass fraction purity of more than 0.98. DMF was obtained from Sigma-Aldrich South Africa and has a mass fraction purity of 0.998. Ultrasound degassing and vacuum drying were performed to dry the ILs before use. The water content in the ILs and DMF was assessed using a Karl–Fischer Titrator (Metrohm 756) and found to be less than 0.03%. Table 1 has a detailed summary of all the chemicals used in this study.

**Table 1:** Specifications of chemicals

Chemicals	CAS No.	Source	Molar mass (g mol^−1^)	Purification method	Purity^a^ mass fraction
[Bmim][SCN]	344790-87-0	IoLiTec, Germany	197.30	No further purification	>0.98
[Bmim][Cl]	79917-90-1	IoLiTec, Germany	174.67	No further purification	0.99
[Bmpym][Cl]	479500-35-1	IoLiTec, Germany	177.72	No further purification	0.99
DMF	68-12-2	Sigma-Aldrich	73.09	No further purification	0.998

a As declared by the supplier.

### 2.2 Density and sound velocity measurements

The binary mixtures were prepared by transferring the pure ILs and DMF, using a syringe (to prevent contamination and evaporation), into stoppered glass vials. DMF was added first into an air-tight stoppered 10 cm^3^ glass vial and weighed. The IL was then added into the same glass vial and weighed again. The uncertainty in the molality (*m*) was estimated to be less than 0.0007 mol kg^−1^. The Anton Paar DSA-5000M densitometer and sound velocity analyser (temperature range from (273.15–343.15) K), with an uncertainty of 0.94 kg m^−3^ for density and 2.9 m s^−1^ for sound velocity, was used to record the density and sound velocity for all the pure compounds and their binary mixtures. The ILs [Bmim][Cl] and [Bmim][SCN] used in this study have melting points between (343.15–353.15) K but [Bmpym][Cl] has a melting point of 471.15 K. Hence, the temperature of (293.15–343.15) K was chosen. The details for the procedure, used in this study, for recording density and speed velocity on the DSA 5000M densitometer is reported in our previous publication.^25^ The uncertainties in *V*_ϕ_ and *K*_ϕ_ are 0.009 cm^3^ mol^−1^, and 0.08 × 10^−5^ cm^3^ mol^−1^ Pa^−1^, respectively. To ensure the reproducibility and accuracy of the measurement, each sample was analysed in triplicate and the average of the data was reported with their uncertainties. Deionized water was used for the calibration.

### 2.3 DFT calculations

GaussView 6.0.16 was used to draw the 3D structures (Fig. S1, ESI†) of the ionic liquids.^26^ All calculations in this investigation were performed using the 6-311++G(d,p) basis set^27^ and the B3LYP-D3 functional.^28^ Diffuse and polarized functions are required in the basis set for systems that comprise both cations and anions. All of the computations were performed using Gaussian 16.^29^ (The *XYZ* coordinates of the optimised IL complexes (with one solvent molecule) in the gas phase is given in the ESI†) The interaction energy (*E*_inter_) was estimated using eqn (1)

1 *E*_inter_ = *E*_ILcomplex_ − *E*_IL–anion_ − *E*_IL–cation_ − *E*_solvent molecule_

In this study, the total energy of the complex (comprising IL, anion/cation, and solvent molecule) is *E*_ILcomplex_. *E*_IL–anion_ is the energy of the anion in the ionic liquid, *E*_IL–cation_ is the energy of the cation in the ionic liquid and *E*_solvent molecule_ is the energy of the isolated solvent molecule. The gas phase interaction energy was corrected using Boys and Bernardi's^30^ counterpoise (CP) correction which is accepted as a reliable technique for taking basis set superimposition error (BSSE) into account. The SMD solvation model^31^ was used to calculate the IL–solvent interactions in the presence of dimethyl formamide (DMF, dielectric constant (*ε*) of 37.51 at 298.15 K) (Table S2, ESI†).

The quantum theory of atoms in molecules (QTAIM) study was done to get an understanding of the type of interaction that occurs between the ionic liquid and one solvent molecule.^32^ A reduced density gradient (RDG) plot was created using the Multiwfn program^33^ and non-covalent interaction (NCI) analysis was carried out using the following formula:
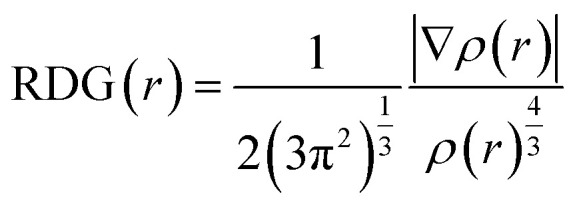
where, *ρ*(*r*) is the quantum-mechanical electron density and ∇*ρ*(*r*) is electron density gradient, which measures the rate at which the electron density changes in space. The type of interaction—hydrogen bonding, steric interactions, or van der Waals's interaction—is depicted in this analysis using colour coding.^34^

## 3. Results and discussion

### 3.1 Experimental

**Fig. 1:**
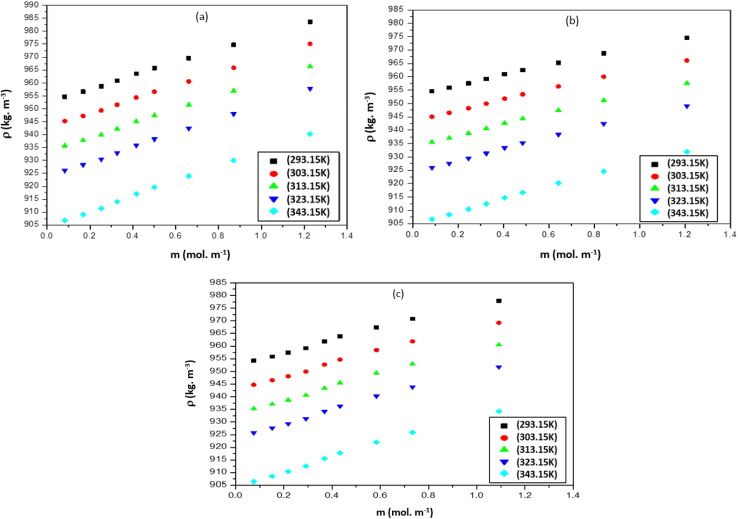
Density (*ρ*), for the mixtures of (a) [Bmim][Cl] + DMF, (b) [Bmpym][Cl] + DMF and (c) [Bmim][SCN] + DMF.

Density (*ρ*) and sound speed (*u*) were measured to analyze the molecular interactions of polar solvent DMF with various ILs. The properties of [Bmim][Cl], [Bmpym][Cl] and [Bmim][SCN] with DMF at different temperatures and at ambient pressure are reported in this work. The experimental *ρ* and *u* values for three ILs with DMF are presented in Table S1 (ESI†) and graphically presented in Fig. 1.

It has been established that ILs are miscible with liquids possessing a medium-to-high dielectric constant, but are immiscible with those having a low dielectric constant. In this investigation, all ILs were shown to be completely miscible in DMF due to the high DMF dielectric constant value.

The dielectric constant value for DMF, [Bmim][Cl], [Bmpym][Cl] and [Bmim][SCN] are approximately 37.5, 15.1, 13.4 and 16.2 at 298.15 K, respectively. Since these are approximate values, they can vary depending on the specific conditions and chemical purity. Our results show that the *ρ* values for all the binary mixtures increase with an increase in concentration of the ILs in DMF over the investigated temperature range. This follows the trend reported in literature^35^ for these systems [C_*n*_mim]Br (*n*) 8, 10, 12) in DMF at temperature 298.15 K. It was also observed that the *ρ* values decrease with increasing temperature.

**Fig. 2:**
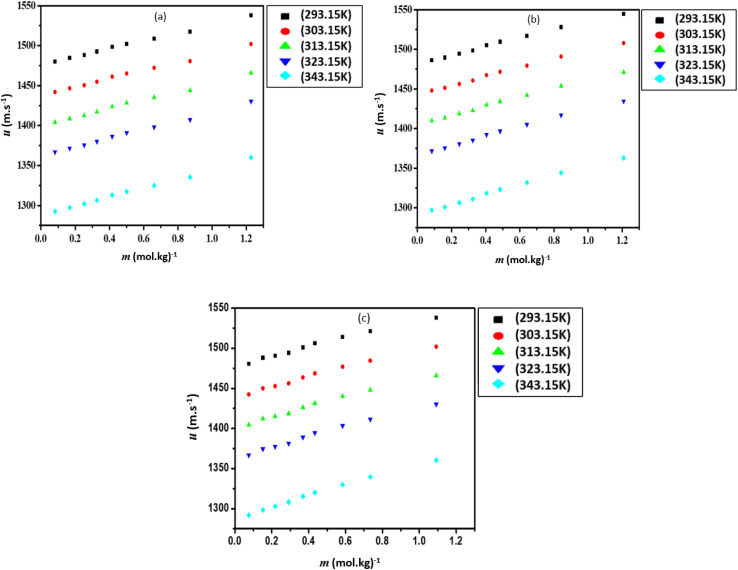
Sound velocity (*u*), for the mixtures of (a) [Bmim][Cl] + DMF, (b) [Bmpym][Cl] + DMF and (c) [Bmim][SCN] + DMF.

The *ρ* values of three ILs with DMF vary at high temperatures. The mixture of [Bmim][Cl] and [Bmim][SCN] with DMF have similar *ρ* values but for [Bmpym][Cl]–DMF mixture there is a slight decrease in the *ρ* values. This may be due to the progressive structural effect of DMF on the imidazolium and pyrrolidinium cation. The *u* values for the mixtures of ILs with DMF at different temperatures are presented in Table S3 (ESI†) and shown graphically in Fig. 2. The data shows that, at each temperature, the *u* values increase with an increase in IL concentration. However, the *u* values decrease as the temperature increases.

**Fig. 3:**
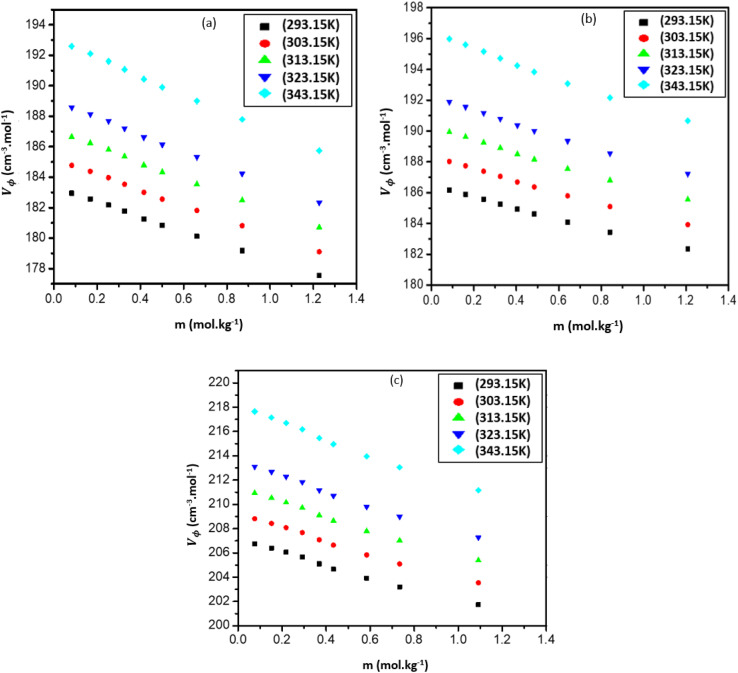
Apparent molar volume (*V*_ϕ_), for the mixtures of (a) [Bmim][Cl] + DMF, (b) [Bmpym][Cl] + DMF and (c) [Bmim][SCN] + DMF.

Apparent molar volumes (*V*_ϕ_) was calculated using eqn (2),2
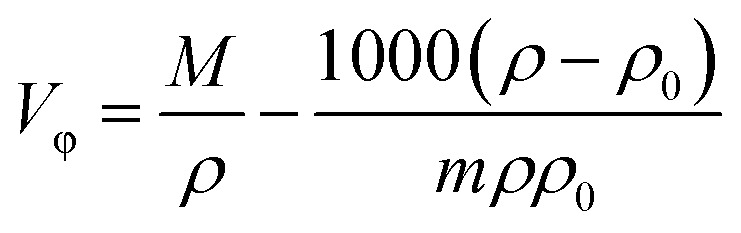
where *M* is the molar mass of the IL, *m* is the molality of the IL, *ρ*_0_ and *ρ* is the density of the pure liquid and the mixture, respectively. The calculated *V*_ϕ_ values are presented in Table S3 (ESI†) and shown graphically in Fig. 3.

In Fig. 3 it can be seen that all three IL mixtures: [Bmim][Cl], [Bmpym][Cl] and [Bmim][SCN] + DMF have positive (*V*_ϕ_) values that increase as the temperature increases but decrease with a rise in IL concentration. The apparent molar volume values for [Bmim][SCN] are higher than the values obtained for [Bmpym][Cl] and [Bmim][Cl]. This could be attributed to several factors, such as the molecular interactions and structural differences as well as the effect of cation and anion. The size and structure of the ions in the ILs can also affect the apparent molar volumes. [Bmim][SCN] has a larger and more complex molecular structure when compared to [Bmim][Cl] and [Bmpym][Cl. This large size and increased molecular complexity of [Bmim][SCN] may result in a higher apparent molar volume due to increased steric hindrance. The nature of the interactions between the ions in the ILs can influence apparent molar volumes. The thiocyanate anion (SCN^−^) in [Bmim][SCN] may lead to stronger ion–ion interactions than the interactions between the chloride anion (Cl^−^) in both [Bmim][Cl] and [Bmpym][Cl]. These strong ion–ion interactions can expand the IL structure, resulting in a higher apparent molar volume. Also, the choice of solvent, in this study, DMF, can have an impact the apparent molar volumes. As already stated, DMF is a polar solvent and can interact with the ions in the ILs *via* dipole–dipole interactions or hydrogen bonding. Furthermore, it should be noted that specific experimental conditions, such as varying temperature and pressure, can also influence the apparent molar volumes. Hence, the observed differences in the apparent molar volumes for the [Bmim][SCN], [Bmim][Cl], and [Bmpym][Cl] mixtures in DMF could be a combination of these factors. To obtain a precise understanding, more experimental investigation, which is beyond the scope of this investigation, and further analysis is required.

The limiting apparent molar volumes for the binary mixtures are essential because it gives valuable information on the solute–solvent interactions. The apparent molar volume values are fitted to the Redlich–Meyer equation (eqn (3)),

3 *V*_ϕ_ = *V*^0^_ϕ_ + *S*_v_*m*^1/2^ + *B*_v_*m*

where *V*^0^_ϕ_ is the limiting apparent molar volume, *S*_v_ and *B*_v_ are empirical parameters.

The *V*^0^_ϕ_, *S*_v_ and *B*_v_ values are presented in Table 2. The *V*^0^_ϕ_ values of ILs represents the limiting value of their apparent molar volume as concentration approaches zero. At this boundary, the ions of the ILs are surrounded only by the DMF solvent, while the other ions are at an infinite distance. Hence, this is a just a measurement of the interaction between the ions and the solvent. The *V*^0^_ϕ_ values (Table 2) are positive and increases as the temperature increases, for all the systems under investigation. This observation is consistent with that reported in literature^35–38^ for the systems [C_*n*_mim]Br (*n*) 8, 10, 12) or [MMIm][MSO_4]_ or DEA/TfO or TEA/TfO or TetEA/TfO or [TMG][Oct] or [TMG][Dec] or [TMG][Lab] with DMF. The *V*^0^_ϕ_ values follow the trend: [Bmim][Cl]< [Bmpym][Cl]< [Bmim][SCN] indicating that both the cation and anion of the IL play a key role on ion–DMF interactions. The *V*^0^_ϕ_ values obtained in this study indicate that the ion–DMF interactions of [Bmim][SCN] system are stronger than those of the [Bmpym][Cl] system and the [Bmim][Cl] system. The high ionic radius of the [SCN] anion as compared to [Cl] anion also influences the strong ion–DMF interactions in the [Bmim][SCN] system. The [Bmim][Cl] and [Bmpym][Cl] systems show a similar effect of the cation on ion–DMF interactions, indicating the cation effect on the structure.

**Table 2:** The values of *V*^0^_ϕ_, *S*_v_, *B*_v_ and standard deviation (*σ*) for (ILs + DMF) at different temperatures^a^

*T* (K)	*V* ^0^_ϕ_ (cm^3^ mol^−1^)	*S*_v_ (cm^3^ mol^−3/2^ kg^1/2^)	*B*_v_ (cm^3^ mol^−2^ kg)	*σ*
**[Bmim][Cl] + DMF**				
293.15	183.596	−0.911	−4.114	0.05
303.15	185.441	−0.890	−4.372	0.05
313.15	187.360	−0.972	−4.558	0.05
323.15	189.319	−1.025	−4.771	0.06
343.15	193.440	−1.249	−5.160	0.06

**[Bmpym][Cl] + DMF**				
293.15	186.839	−1.454	−2.429	0.04
303.15	188.757	−1.586	−2.587	0.05
313.15	190.721	−1.716	−2.748	0.05
323.15	192.732	−1.830	−2.930	0.05
343.15	196.930	−2.665	−3.348	0.06

**[Bmim][SCN] + DMF**				
293.15	207.762	−2.332	−3.366	0.12
303.15	209.879	−2.454	−3.534	0.12
313.15	212.057	−2.629	−3.673	0.12
323.15	214.302	−2.832	−3.813	0.13
343.15	219.004	−3.321	−4.099	0.14

a Standard uncertainties *u* is *u*(*ρ*) = 0.94 kg m^−3^, *u*(*u*) = 2.9 m s^−1^; *u*(*m*) = 0.0007 mol kg^−1^; *u*(*T*) = 0.001 K, *u*(*p*) = 0.01 MPa, *u*(*V*_ϕ_) = 0.009 cm^3^ mol^−1^ and *u*(*K*_ϕ_) = 0.08 × 10^−5^ cm^3^ mol^−1^ Pa^−1^ (0.68 level of confidence).

As reported in literature^6,39–41^ the *S*_v_ values are generally positive but for some electrolytes it can be negative.^42–44^ In this study, the *S*_v_ values are negative for all three ILs binary mixtures across the temperature range under investigation. This can be attributed to various factors related to the ion–DMF interactions and the structural changes within the mixtures. The nature and strength of the ion–DMF interactions between the ILs and DMF can influence the *S*_v_ values. The negative *S*_v_ values clearly indicate that the ion–DMF interactions is stronger than the (ion–ion) interactions of IL cations and anions. The same trend was also reported^45^ for the system IL BDMAH with DMF. The *S*_v_ values decrease with an increase of temperature with the exception of the [Bmim][Cl] system at 303.15 K.

*B*_v_ values are typically negative except in the case of hydrogen bonding interactions.^46^ In Table 3, the *B*_v_ values are negative for all three systems. The negative *B*_v_ values may be due to presence of DMF molecules which increases the co-sphere overlap of the solute ILs [Bmim][SCN], [Bmim][Cl], and [Bmpym][Cl. A similar trend was observed for the following reported IL systems: [Emim][NTf_2_] + DEC or PEGMME,^46^ [Bmmim][NTf_2_] + DEC or PEGMME^46^ systems. These systems: [Emim][NTf_2_] or [Bmim][NTf_2_ ] or [Bmmim][NTf_2_] + DMC,^6^ [MOEmmim][NTf_2_] + DEGDME,^24^ BDMAP + DMF,^45^ [CyN1, 1PrSO_3_ H][Tos] + methanol,^47^ [Bmim][TfO] + alcohol,^48^ [MOA][NTf_2_] + ethyl acetate^49^ also have negative *B*_v_ values. Negative *B*v values for the BDMAP system with DMF were also reported by Keshapolla *et al.* and Makarov *et al.*^37,45^

**Table 3:** The *E*^0^_ϕ_ and isobaric thermal expansion coefficients (*α*_p_)^a^

Solute	Solvent	293.15 K	303.15 K	313.15 K	323.15 K	343.15 K
***E*** ^**0**^_**ϕ**_ **(cm** ^**3**^ **mol^−^** ^**1**^ **K^−^** ^**1**^ **)**						
[Bmim][Cl]	DMF	0.182	0.188	0.194	0.200	0.212
[Bmpym][Cl]	DMF	0.189	0.194	0.199	0.204	0.215
[Bmim][SCN]	DMF	0.208	0.215	0.221	0.228	0.242

**10^−^** ^**3**^ **× *α***_**p**_ **(K^−^** ^**1**^ **)**						
[Bmim][Cl]	DMF	0.99	1.01	1.04	1.06	1.10
[Bmpym][Cl]	DMF	1.01	1.03	1.04	1.06	1.09
[Bmim][SCN]	DMF	1.00	1.02	1.04	1.06	1.11

a Standard uncertainties *u* is *u*(*ρ*) = 0.94 kg m^−3^, *u*(*u*) = 2.9 m s^−1^; *u*(*m*) = 0.0007 mol kg^−1^; *u*(*T*) = 0.001 K, *u*(*p*) = 0.01 MPa, *u*(*V*_ϕ_) = 0.009 cm^3^ mol^−1^ and *u*(*K*_ϕ_) = 0.08 × 10^−5^ cm^3^ mol^−1^ Pa^−1^ (0.68 level of confidence).

The temperature dependence of *V*^0^_ϕ_ is expressed as a second-order polynomial of absolute temperature:^39^

4 *V*^0^_ϕ_ = *A* + *BT* + *CT*^2^

where *A*, *B* and *C* are empirical parameters and *T* is the temperature. The limiting apparent molar expansibility *E*^0^_ϕ_ is obtained by differentiating eqn (4) with respect to temperature:5
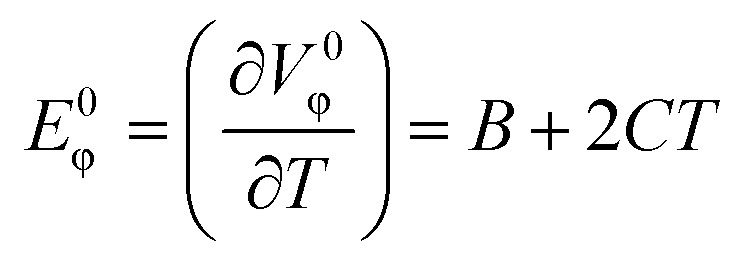


The *E*^0^_ϕ_ value presents insights into the dynamics of IL–DMF interactions. The values of *E*^0^_ϕ_ for the studied ILs are given in Table 3 as a function of temperature. The *E*^0^_ϕ_ values are positive for all the systems. This result is similar to that reported in the literature^46^ for the ILs BDMAH or BDMAP system with DMF. Positive *E*^0^_ϕ_ values were also reported for the [TMG][Oct] or [TMG][Dec] or [TMG][Lab] system with DMF.^38^ The positive *E*^0^_ϕ_ values may be the result of the pure DMF expanding more rapidly than the solution containing the IL ions. In addition, *E*^0^_ϕ_ increases steadily when the temperatures rise. This could be as a result of DMF molecules being liberated from IL ions at higher temperatures which would increase the volume of the (ILs + DMF) solution more than it would for pure DMF.

The solute's *α*_p_, was calculated using eqn (6):6
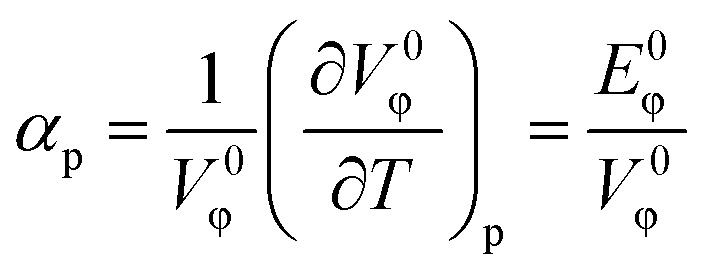


The calculated *α*_p_ describes the effect of temperature on volume of the (IL + DMF) solution while maintaining a constant pressure. A positive *α*_p_ was obtained for all the systems and it was observed that *α*_p_ increases as the temperature increased (Table 3).

**Fig. 4:**
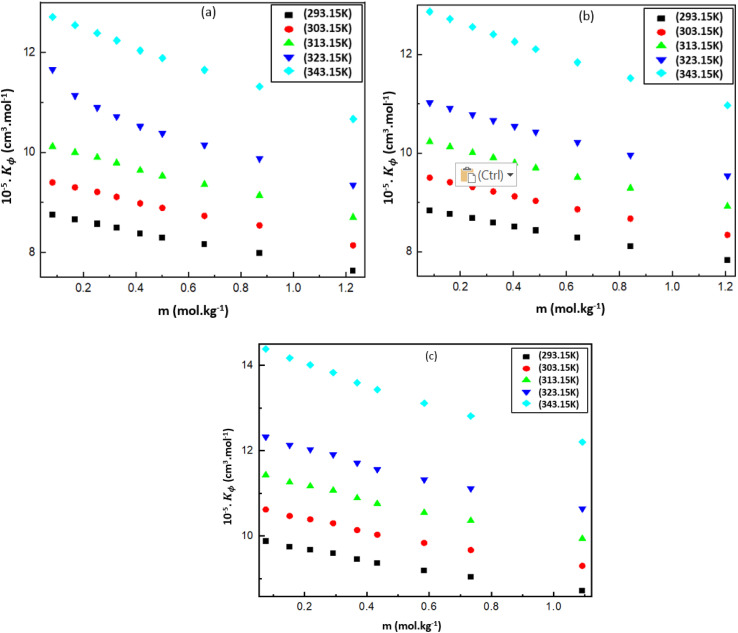
Apparent molar adiabatic compressibility (*K*_ϕ_), for the mixtures of (a) [Bmim][Cl] + DMF, (b) [Bmpym][Cl] + DMF and (c) [Bmim][SCN] + DMF.

The apparent molar adiabatic compressibility (*K*_ϕ_) values, at various temperatures, were calculated for the [Bmim][Cl] + DMF, [Bmpym][Cl] + DMF and [Bmim][SCN] + DMF systems, using eqn (7):7
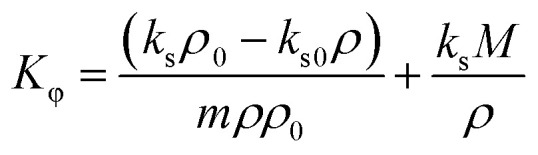
where *k*_s_ and *k*_s0_ are the isentropic compressibilities of solution and pure solvent, respectively. The (*K*_ϕ_) values are presented Table S3 (ESI†) and is displayed in the Fig. 4.

The values for these mixtures are positive and increase with increasing temperature but decrease with increasing molality of the IL in the DMF. This can be attributed to several factors related to the molecular interactions and structural changes within the mixtures (Fig. 4). The positive *K*_ϕ_ values indicate that there is a weak interaction between the DMF and IL. Similar results are reported in literature^38,45^ for the ILs BDMAH or BDMAP or [TMG][Oct] or [TMG][Dec] or [TMG][Lab] system with DMF.

Limiting apparent molar adiabatic compressibility (*K*^0^_ϕ_), values were computed using the standard Redlich–Mayer eqn (8):

8 *K*_ϕ_ = *K*^0^_ϕ_ + *S*_k_*m*^1/2^ + *B*_k_*m*

The calculated *K*^0^_ϕ_, *S*_k_ and *B*_k_, values for each mixture at the given experimental temperature are summarized in Table 4.

**Table 4:** The values of *K*^0^_ϕ_, *S*_k_, *B*_k_, and *σ* for (ILs + DMF) at different temperatures^a^

*T* (K)	10^−5^ × *K*^0^_ϕ_ (m^3^ mol^−1^ Pa^−1^)	10^−5^ × *S*_k_ (m^3^ mol^−3/2^ kg^1/2^ Pa^−1^)	10^−5^ × *B*_k_ (m^3^ mol^−2^ kg Pa^−1^)	10^−5^ × *σ*
**[Bmim][Cl] + DMF**				
293.15	8.91	−0.325	−0.743	0.01
303.15	9.570	−0.330	−0.864	0.02
313.15	10.331	−0.440	−0.926	0.02
323.15	12.520	−3.458	−0.577	0.06
343.15	12.967	−0.454	−1.448	0.02

**[Bmpym][Cl] + DMF**				
293.15	9.011	−0.390	−0.632	0.01
303.15	9.712	−0.484	−0.701	0.01
313.15	10.470	−0.556	−0.786	0.01
323.15	11.275	−0558	−0.937	0.01
343.15	13.187	−0.690	−1.216	0.02

**[Bmim][SCN] + DMF**				
293.15	10.167	−0.841	−0.535	0.02
303.15	10.940	−0.940	−0.616	0.03
313.15	11.791	−1.061	−0655	0.03
323.15	12.739	−1.240	−0.755	0.04
343.15	14.913	−1.528	−1.045	0.04

a Standard uncertainties *u* is *u*(*ρ*) = 0.94 kg m^−3^, *u*(*u*) = 2.9 m s^−1^; *u*(*m*) = 0.0007 mol kg^−1^; *u*(*T*) = 0.001 K, *u*(*p*) = 0.01 MPa, *u*(*V*_ϕ_) = 0.009 cm^3^ mol^−1^ and *u*(*K*_ϕ_) = 0.08 × 10^−5^ cm^3^ mol^−1^ Pa^−1^ (0.68 level of confidence).

It was observed (Table 4) that the *K*^0^_ϕ_ values are positive and increase with an increase in temperature for all studied binary systems. The positive values are reported for DBU based ILs in DMF^50^ and [TMG][Oct] or [TMG][Dec] or [TMG][Lab] with DMF.^38^ This could be because (IL + DMF) solution more compressible due to the intrinsic compressibility of the DMF caused by the intermolecular free space. The positive values may be due to the increased compressibility of the (ILs + DMF) solution in comparison to pure DMF.^49^ Empirical parameters *S*_k_ and *B*_k_ have the same significance as *S*_v_ and *B*_v_. The *K*^0^_ϕ_ values follow the trend: [Bmim][Cl] < [Bmpym][Cl] < [Bmim][SCN] indicating that both cation and anion of the IL play a key role on *K*^0^_ϕ_ values. Thus, the *K*^0^_ϕ_ values obtained in this study indicate that the [Bmim][SCN] system has a value than those of [Bmpym][Cl] system and [Bmim][Cl] system. The higher *K*^0^_ϕ_ values for the [Bmim][SCN] system indicate that the [SCN] anion has high ionic radius than the [Cl] anion, demonstrating the impact of the anion on *K*^0^_ϕ_ values. The [Bmim][Cl] and [Bmpym][Cl] systems show a similar effect of the cation on *K*^0^_ϕ_ values indicating the influence of cation structure on *K*^0^_ϕ_.

### 3.2 DFT calculations

**Fig. 5:**
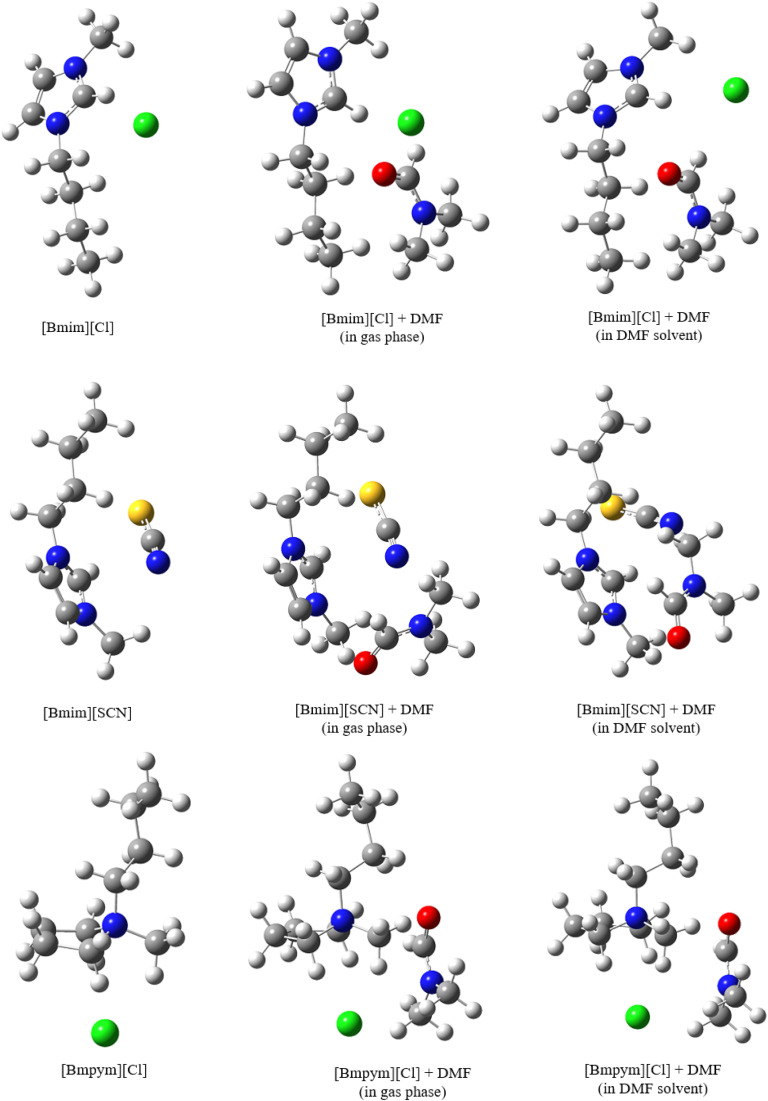
Optimised structures for the isolated ILs and (ILs + DMF) mixture (in gas phase and in solvent) as calculated at 298.15 K.

As mentioned, one of the aims of this study was to determine the type of interaction between the ions in the IL. One DMF molecule was introduced, creating the IL–solvent complexes (Fig. 5) to simulate bonding between the optimized ILs and the solvent. The complex was optimized in the gas phase and in DMF solvent. Since there is no solvent interference, the interaction energies in gas phase are lower for both the corrected and uncorrected energies (Table 5). The interaction energies are much higher in the solvent due to the IL complex–solvent interactions. The data shows that [Bmim][Cl] has the lowest interaction energy indicating weak interactions in solvent.

**Table 5:** Calculated interaction energy for the IL complexes^a^

Structure	Δ*E*_inter_ (kcal mol^−1^)		
	Gas phase – uncorrected	Gas phase – corrected	Solvent–DMF
[Bmin][SCN]-DMF	−103.14	−108.39	−6.0806
[Bmin][Cl]-DMF	−108.74	−119.48	−4.9586
[Bmpym][Cl]-DMF	−108.85	−130.30	−5.5635

a The calculated interaction energies indicate the [Bmim][SCN]–DMF system is the most efficient system due to its lowest interaction energy. This conclusion aligns with the observations from the experimental data.

### 3.3 QTAIM and NCI calculations

To determine the type of interaction the QTAIM study and NCI analysis was done in both the gas phase and in solvent. In the discussion below, we have presented only the data from the solvent interactions but we have referred to the results from the gas phase which is presented (in detail) in the ESI.† For the [Bmim][Cl]–DMF complex in the gas phase the ionic liquid interacts with the DMF molecule through four specific interactions (Fig. S1a†). According to the quantum theory of atoms in molecules (QTAIM), the typical range of electron density (*ρ*) for classical hydrogen bonds lies between 0.002 and 0.035 a.u. The electron density value serves as an important measure of the strength of non-covalent interactions between atoms. Lower values (near 0.002 a.u.) indicate weak interactions such as van der Waals forces or very weak hydrogen bonds. On the other hand, as the electron density approaches 0.035 a.u., it signals a stronger hydrogen bond, indicating a more significant interaction between the donor and acceptor atoms.^51^ The sign of Laplacian of electron density, ∇^2^*ρ*(*r*) describes the characteristics of the bond: it is non-covalent (closed-shell) bonding, for example ionic, hydrogen bonding or van der Waals interaction if ∇^2^*ρ*(*r*) > 0. It is covalent if ∇^2^*ρ*(*r*) < 0. There is clear indication of non-covalent interaction between the anion of the ionic liquid and the cation (Fig. S1b and c†). When the [Bmim][Cl]–DMF complex is added to DMF solvent, an increase in bond lengths (between the bonding atoms) is observed and there are now only two possible interactions (Fig. S2†). This is expected since the complex is in solvent. The localized orbital locator, LOL(*r*) which is used to identify regions of high electron localization was proposed by Schmider and Becke.^51^ When LOL(*r*) > 0.5 a.u. it denotes shared interactions or localized orbitals.

**Fig. 6:**
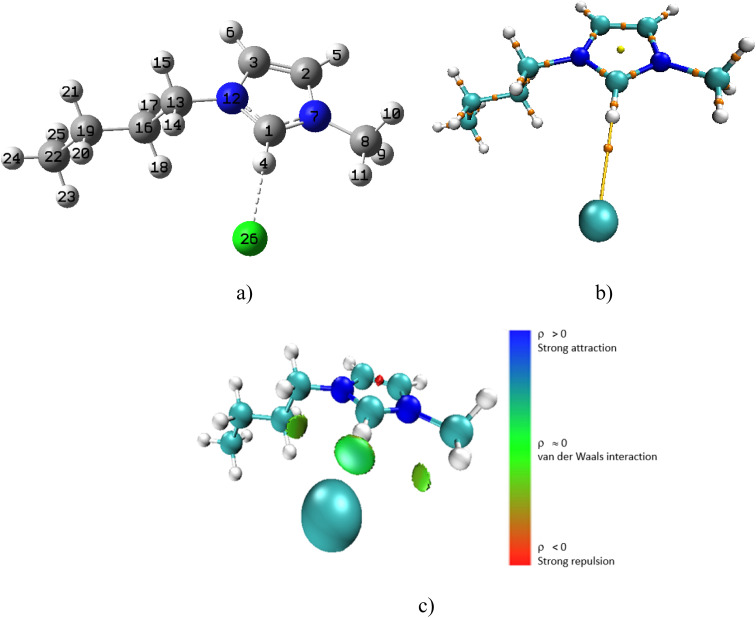
(a) Optimized [Bmim][Cl] complex in DMF solvent (b) molecular graph with BCPs and (c) 3D NCI plot.

When the [Bmim][Cl] complex was modelled alone in DMF solvent, a weak interaction between the chloride ion and the H4 atom of the cation was observed (Fig. 6a). This weak interaction is confirmed by the appearance of a bond critical point (BCP) between H4 and the chloride anion in the molecular graph (Fig. 6b) and the observed light blue isosurface in the NCI plot (Fig. 6c). The electron density, *ρ*(*r*), at this BCP is 0.0015 a.u., and the Laplacian of the electron density, ∇^2^*ρ*(*r*), is positive with a value of 0.0046 a.u. Although the bond length between the ions has increased in solvent, there is still weak interaction between Cl^−^ and H4 and as indicated (Table 5), this complex has the highest interaction energy in solvent.

**Fig. 7:**
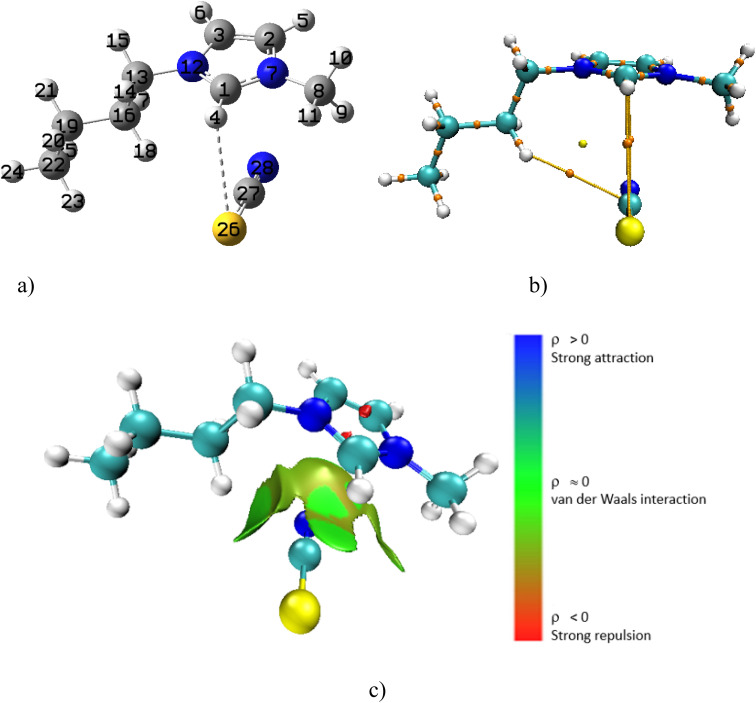
(a) Optimized [Bmim][SCN] complex in DMF solvent (b) molecular graph with BCPs and (c) 3D NCI plot.

For the [Bmim][SCN]–DMF complex in the gas phase, there are also four bond critical points (BCPs) in the molecular graph (Fig. S3a and b†). The green isosurfaces (Fig. S3c†) between the interacting atoms in the [Bmim][SCN]–DMF complex confirms the presence of weak interactions. However, the *ρ*(*r*) value for O39–H9 for the DMF molecule is largest (0.0133 a.u.) (Table S5†), suggesting that this is the strongest interaction in this IL–DMF interaction. When the [Bmim][SCN]–DMF complex is added to DMF solvent (Fig. S4†), two interactions disappeared. Although, here as well, the bond lengths between the interacting atoms have increased, there is still weak interaction. Next, the [Bmim][SCN] complex was optimized in DMF only (Fig. 7a). The 3D NCI plot, molecular graph, as well as the optimized structure reveals that the cation interacts specifically through the SCN ion with S26 and H4 of the cation *via* S26–H4 contact (Fig. 7b). The positive ∇^2^*ρ*(*r*) value (0.0179 a.u.), indicates that the C27–H4–S26 interaction (bond distance 3.16 Å) is non-covalent. The low *ρ*(*r*) value at the bond critical point (0.0085 a.u.) verifies the weak non-covalent C27–H4–S26 interaction. The presence of green isosurfaces between these atoms in the 3D NCI plot (Fig. 7c) confirms that the interactions between the cation and anion are weak and non-covalent in nature. A comparison of the electron density, *ρ*(*r*) and the Laplacian of the electron density, ∇^2^*ρ*(*r*) at the BCP for [Bmim][Cl] and [Bmim][SCN] in solvent was done. For [Bmim][Cl] the interaction in solvent was between H4–Cl^−^ with a *ρ*(*r*) value of 0.0015 a.u. and a positive ∇^2^*ρ*(*r*) of 0.0046 a.u. The *ρ*(*r*) and ∇^2^*ρ*(*r*) for [Bmim][SCN] in solvent was higher. As indicated in the discussion above, for [Bmim][SCN] the only interaction in solvent was *via* S26–H4 with a *ρ*(*r*) value of 0.0085 a.u. and a positive ∇^2^*ρ*(*r*) of 0.0179 a.u. These higher values in the [Bmim][SCN] complex aligns with what was observed experimentally. Also, as indicated (Table 5), this complex has the lowest interaction energy in solvent thus making it the favoured system in this solvent.

**Fig. 8:**
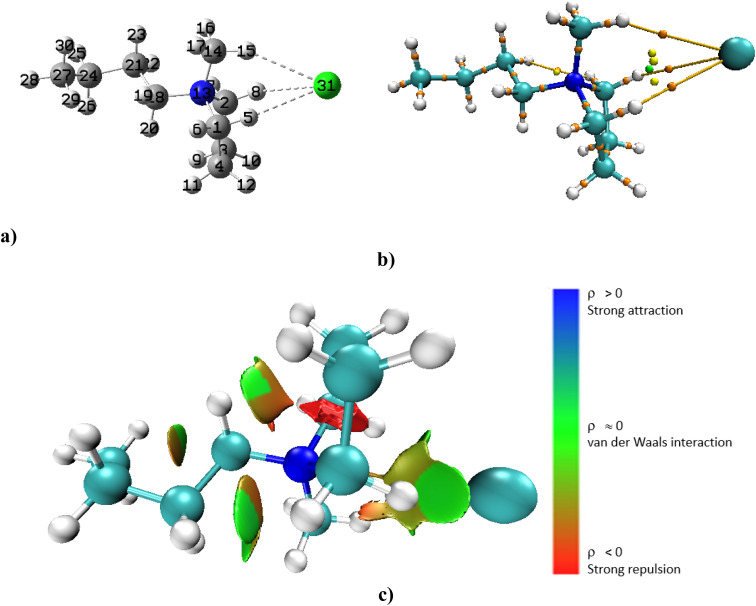
(a) Optimized [Bmpym][Cl] complex in DMF solvent (b) molecular graph with BCPs and (c) 3D NCI plot.

For the Bmpym][Cl]–DMF complex in the gas phase, five non-covalent interactions between DMF and the ionic liquid was observed (Fig. S5a and b†). The calculated ∇^2^*ρ*(*r*) values at these BCPs are positive, indicating that these interactions are non-covalent (Table S7†). However, when the [Bmpym][Cl]–DMF complex is in solvent it shows only two weak interactions (Fig. S6†) indicating the weak van der Waals interactions. The optimized [Bmpym][Cl] complex was then placed in DMF solvent and the output shows that the chloride anion interacts with the cation *via* H5–Cl^−^, H15–Cl^−^ and H8–Cl^−^ (Fig. 8a). The green isosurfaces observed between the hydrogen atoms and the chloride anion confirm that these interactions are van der Waals in nature (Fig. 8c). The molecular graph reveals three BCPs: (Fig. 8b). The *ρ*(*r*) and ∇^2^*ρ*(*r*) values at these BCPs were as follows: H5–Cl31 (*ρ*(*r*) is 0.0034 a.u. and ∇^2^*ρ*(*r*) is 0.0140 a.u.); H15–Cl31 (*ρ*(*r*) is 0.0031 a.u. and ∇^2^*ρ*(*r*) is 0.0132 a.u.) and H8–Cl31 (*ρ*(*r*) is 0.0033 and ∇^2^*ρ*(*r*) is 0.0135 a.u.). The positive ∇^2^*ρ*(*r*) value for all the BCPs indicate the interactions are non-covalent in nature. However, all the *ρ*(*r*) values less than 0.01 a.u. suggesting that the interaction between the chloride anion and the hydrogen atoms of the cation is weak. A comparison of the data from [Bmpym][Cl] and [Bmim][Cl] complexes was done. The difference between the two systems is one nitrogen atom in [Bmim] is replaced with a carbon atom in [Bmpym]. The *ρ*(*r*) and ∇^2^*ρ*(*r*) values in solvent, are higher for [Bmpym][Cl] than the values that were observed for the [Bmim][Cl] system which may be the reason for its high interaction energy.

The electron density values which serve as an important measure of the strength of non-covalent interactions between atoms was very important in this study. We noted that although, the simulation with one DMF molecule did give us an understanding of how the IL interacts in the solvent, the electron density revealed that all three systems have weak noncovalent interaction. When the IL complex was modelled only in DMF solvent (as discussed above), there was a difference in the electron density compared to the systems that included one DMF molecule. [Bmim][Cl] by itself, in solvent had an electron density of 0.0046 a.u.; [Bmim][SCN] in solvent was 0.0085 a.u. and [Bmpym][Cl] was 0.0034 a.u. Hence, the QTAIM and NCI data, complemented the experimental observation: [Bmim][SCN] is the most efficient system in this solvent.

## 4. Conclusion

The *V*_ϕ_ and *K*_ϕ_ values as a function of IL concentration were calculated using the newly determined experimental *ρ* and *u* to assess the impact of the cation/anion of ILs on solute–solute, solute–solvent interactions between DMF and ILs [Bmim][Cl], [Bmpym][Cl], [Bmim][SCN]. The Redlich–Mayer type equation was used to evaluate the corresponding infinite dilution values of *V*_ϕ_ and *K*_ϕ_ together with empirical parameters. The *V*^0^_ϕ_ values for the [Bmim][SCN] system are higher than those of the [Bmim][Cl] system which may be because the [SCN] anion has a larger ionic radius compared to the [Cl] anion. This indicates the influence of the anion on ion–DMF interactions. For the mixtures in this study, negative *S*_v_ values indicate that the ion–DMF interactions are stronger than the (ion–ion) interactions. The intermolecular free space, which increases the compressibility of the (IL + DMF), is thought to be the cause of the positive values of *K*^0^_ϕ_. The results reported in this study are similar to published reports.^6,35–50^ The calculated interaction energies for each complex were further investigated by QTAIM and NCI analysis. The results from these calculations reveal the presence of weak van der Waals interactions in all three complexes, and confirmed the experimental observation that the [Bmim][SCN] system is the most effective complex in this solvent.

## Data availability

The data supporting this article have been included as part of the ESI.†

## Conflicts of interest

The authors declare no conflict of interest.

## Supplementary Material

RA-015-D4RA08020C-s001
