# Supplementary material for: An experimental and computational study to explore the ion–solvent interactions between selected ionic liquids and dimethylformamide
Source: RSC Adv. 2025 Apr 11;15(15):11451–64. doi: 10.1039/d4ra08020c (PMC11986807; doi:10.1039/d4ra08020c)
Supplement: RA-015-D4RA08020C-s001 [file RA-015-D4RA08020C-s001.pdf]

Supplementary Information (SI) for RSC Advances.
This journal is © The Royal Society of Chemistry 2025

# SUPPORTING INFORMATION

## An experimental and computational study to explore the ion-solvent interactions between selected ionic liquids and dimethylformamide

Sandeep Kumar[a], Teshome Abute Lelisho[b], Indra Bahadur[c,*] and Thishana Singh[a,*]

[a]School of Chemistry and Physics, University of KwaZulu-Natal, Private Bag X54001, Durban, 4000, South Africa

[b]Department of Chemistry, CNCS, Hawassa University, Hawassa, Ethiopia

[c]Department of Chemistry, Faculty of Natural and Agricultural Sciences, North-West University (Mafikeng Campus), Private Bag X2046, Mmabatho 2735, South Africa

*Corresponding author's email: singht1@ukzn.ac.za; bahadur.indra@nwu.ac.za

**Table S1:** Molality (), , ,  and  for (ILs + DMF) at different temperatures.

| *m* (mol.kg$^{-1}$) | *ρ* (kg.m$^{-3}$) | *u* (m.s$^{-1}$) | (cm$^3$.mol$^{-1}$) | 10$^{-5}$× (cm$^3$.mol$^{-1}$. Pa$^{-1}$) |
|---|---|---|---|---|
| | | **[Bmim][Cl] + DMF** | | |
| | | **T = 293.15 K** | | |
| 0.0823 | 954.67 | 1480.0 | 182.944 | 8.75 |
| 0.1680 | 956.61 | 1484.7 | 182.570 | 8.66 |
| 0.2527 | 958.69 | 1488.4 | 182.173 | 8.57 |
| 0.3267 | 960.83 | 1492.6 | 181.765 | 8.49 |
| 0.4162 | 963.54 | 1498.7 | 181.252 | 8.37 |
| 0.5009 | 965.76 | 1502.1 | 180.836 | 8.29 |
| 0.6612 | 969.62 | 1508.6 | 180.116 | 8.16 |
| 0.8708 | 974.69 | 1517.5 | 179.179 | 7.98 |
| 1.2272 | 983.63 | 1538.1 | 177.550 | 7.63 |
| | | **T = 303.15 K** | | |
| 0.0823 | 945.22 | 1441.9 | 184.772 | 9.40 |
| 0.1680 | 947.21 | 1446.6 | 184.381 | 9.30 |
| 0.2527 | 949.33 | 1450.5 | 183.968 | 9.21 |
| 0.3267 | 951.55 | 1454.8 | 183.537 | 9.11 |
| 0.4162 | 954.34 | 1461.0 | 182.999 | 8.98 |
| 0.5009 | 956.61 | 1465.1 | 182.564 | 8.89 |
| 0.6612 | 960.56 | 1472.3 | 181.814 | 8.73 |
| 0.8708 | 965.87 | 1480.5 | 180.814 | 8.54 |
| 1.2272 | 975.10 | 1501.9 | 179.103 | 8.14 |
| | | **T = 313.15 K** | | |
| 0.0823 | 935.71 | 1404.0 | 186.648 | 10.12 |
| 0.1680 | 937.78 | 1408.6 | 186.234 | 10.00 |
| 0.2527 | 939.90 | 1412.7 | 185.813 | 9.90 |
| 0.3267 | 942.25 | 1417.2 | 185.347 | 9.79 |
| 0.4162 | 945.10 | 1423.6 | 184.786 | 9.64 |
| 0.5009 | 947.44 | 1428.3 | 184.330 | 9.53 |
| 0.6612 | 951.52 | 1435.2 | 183.540 | 9.36 |
| 0.8708 | 956.99 | 1443.9 | 182.491 | 9.14 |
| 1.2272 | 966.44 | 1466.0 | 180.706 | 8.70 |
| | | **T = 323.15 K** | | |
| 0.0823 | 926.14 | 1367.0 | 188.575 | 11.66 |
| 0.1680 | 928.29 | 1371.5 | 188.136 | 11.14 |
| 0.2527 | 930.49 | 1375.7 | 187.690 | 10.90 |
| 0.3267 | 932.90 | 1380.1 | 187.203 | 10.72 |
| 0.4162 | 935.81 | 1386.4 | 186.619 | 10.52 |
| 0.5009 | 938.23 | 1390.8 | 186.138 | 10.38 |
| 0.6612 | 942.38 | 1398.0 | 185.319 | 10.15 |
| 0.8708 | 947.99 | 1407.3 | 184.222 | 9.88 |
| 1.2272 | 957.78 | 1430.3 | 182.339 | 9.35 |
| | | **T = 343.15 K** | | |
| 0.0823 | 906.80 | 1292.7 | 192.594 | 12.71 |

| | | | | |
|---|---|---|---|---|
| 0.1680 | 909.10 | 1297.3 | 192.105 | 12.55 |
| 0.2527 | 911.48 | 1302.0 | 191.602 | 12.39 |
| 0.3267 | 914.00 | 1306.5 | 191.071 | 12.24 |
| 0.4162 | 917.04 | 1312.9 | 190.436 | 12.04 |
| 0.5009 | 919.63 | 1317.2 | 189.900 | 11.89 |
| 0.6612 | 924.01 | 1324.8 | 189.000 | 11.65 |
| 0.8708 | 929.97 | 1335.4 | 187.789 | 11.32 |
| 1.2272 | 940.24 | 1360.1 | 185.738 | 10.67 |
| | | **[Bmpym][Cl] + DMF** | | |
| | | **T = 293.15 K** | | |
| 0.0844 | 954.51 | 1486.0 | 186.162 | 8.83 |
| 0.1607 | 955.88 | 1489.5 | 185.894 | 8.76 |
| 0.2458 | 957.52 | 1494.3 | 185.575 | 8.68 |
| 0.3243 | 959.16 | 1498.4 | 185.256 | 8.59 |
| 0.4048 | 960.89 | 1505.1 | 184.922 | 8.51 |
| 0.4845 | 962.47 | 1509.4 | 184.620 | 8.43 |
| 0.6418 | 965.25 | 1516.9 | 184.088 | 8.28 |
| 0.8415 | 968.70 | 1528.0 | 183.432 | 8.11 |
| 1.2079 | 974.53 | 1544.6 | 182.336 | 7.83 |
| | | **T = 303.15 K** | | |
| 0.0844 | 945.06 | 1447.8 | 188.021 | 9.50 |
| 0.1607 | 946.48 | 1451.3 | 187.740 | 9.41 |
| 0.2458 | 948.22 | 1456.3 | 187.393 | 9.31 |
| 0.3243 | 949.92 | 1460.4 | 187.058 | 9.22 |
| 0.4048 | 951.81 | 1467.3 | 186.686 | 9.12 |
| 0.4845 | 953.43 | 1471.7 | 186.368 | 9.03 |
| 0.6418 | 956.38 | 1479.4 | 185.793 | 8.86 |
| 0.8415 | 960.00 | 1490.7 | 185.094 | 8.67 |
| 1.2079 | 966.12 | 1507.7 | 183.922 | 8.34 |
| | | **T = 313.15 K** | | |
| 0.0844 | 935.57 | 1409.7 | 189.929 | 10.23 |
| 0.1607 | 937.05 | 1413.2 | 189.628 | 10.13 |
| 0.2458 | 938.89 | 1418.3 | 189.255 | 10.01 |
| 0.3243 | 940.62 | 1422.7 | 188.905 | 9.91 |
| 0.4048 | 942.64 | 1429.6 | 188.500 | 9.80 |
| 0.4845 | 944.33 | 1434.1 | 188.162 | 9.70 |
| 0.6418 | 947.43 | 1442.0 | 187.546 | 9.51 |
| 0.8415 | 951.23 | 1453.6 | 186.799 | 9.29 |
| 1.2079 | 957.62 | 1471.0 | 185.553 | 8.92 |
| | | **T = 323.15 K** | | |
| 0.0844 | 926.00 | 1371.8 | 191.889 | 11.02 |
| 0.1607 | 927.57 | 1375.4 | 191.564 | 10.91 |
| 0.2458 | 929.48 | 1380.7 | 191.170 | 10.78 |
| 0.3243 | 931.30 | 1385.2 | 190.794 | 10.66 |
| 0.4048 | 933.39 | 1392.3 | 190.365 | 10.54 |
| 0.4845 | 935.16 | 1396.8 | 190.006 | 10.43 |
| 0.6418 | 938.42 | 1404.9 | 189.346 | 10.22 |
| 0.8415 | 942.39 | 1416.8 | 188.549 | 9.96 |

| | | | | |
|---|---|---|---|---|
| 1.2079 | 949.07 | 1434.6 | 187.222 | 9.54 |
| **T = 343.15 K** | | | | |
| 0.0844 | 906.68 | 1296.9 | 195.976 | 12.87 |
| 0.1607 | 908.40 | 1300.8 | 195.604 | 12.72 |
| 0.2458 | 910.48 | 1306.3 | 195.155 | 12.56 |
| 0.3243 | 912.47 | 1311.0 | 194.729 | 12.41 |
| 0.4048 | 914.73 | 1318.4 | 194.246 | 12.26 |
| 0.4845 | 916.68 | 1323.1 | 193.834 | 12.11 |
| 0.6418 | 920.26 | 1331.7 | 193.080 | 11.84 |
| 0.8415 | 924.60 | 1344.1 | 192.174 | 11.52 |
| 1.2079 | 931.94 | 1362.8 | 190.662 | 10.97 |
| **[Bmim][SCN] + DMF** | | | | |
| **T = 293.15 K** | | | | |
| 0.0752 | 954.24 | 1480.6 | 206.746 | 9.88 |
| 0.1519 | 955.87 | 1488.1 | 206.390 | 9.75 |
| 0.2181 | 957.40 | 1490.7 | 206.058 | 9.68 |
| 0.2916 | 959.17 | 1494.1 | 205.676 | 9.60 |
| 0.3686 | 961.83 | 1501.1 | 205.104 | 9.46 |
| 0.4333 | 963.89 | 1506.2 | 204.665 | 9.36 |
| 0.5840 | 967.41 | 1514.2 | 203.920 | 9.19 |
| 0.7337 | 970.78 | 1521.4 | 203.213 | 9.04 |
| 1.0917 | 977.90 | 1538.1 | 201.735 | 8.72 |
| **T = 303.15 K** | | | | |
| 0.0752 | 944.78 | 1442.4 | 208.815 | 10.62 |
| 0.1519 | 946.49 | 1450.1 | 208.434 | 10.47 |
| 0.2181 | 948.08 | 1452.8 | 208.082 | 10.39 |
| 0.2916 | 949.92 | 1456.3 | 207.678 | 10.30 |
| 0.3686 | 952.65 | 1463.5 | 207.079 | 10.14 |
| 0.4333 | 954.65 | 1468.7 | 206.644 | 10.03 |
| 0.5840 | 958.39 | 1477.1 | 205.838 | 9.84 |
| 0.7337 | 961.86 | 1484.5 | 205.096 | 9.67 |
| 1.0917 | 969.20 | 1501.9 | 203.544 | 9.30 |
| **T = 313.15 K** | | | | |
| 0.0752 | 935.29 | 1404.3 | 210.932 | 11.43 |
| 0.1519 | 937.09 | 1412.1 | 210.523 | 11.26 |
| 0.2181 | 938.73 | 1415.1 | 210.153 | 11.17 |
| 0.2916 | 940.64 | 1418.7 | 209.725 | 11.07 |
| 0.3686 | 943.44 | 1426.2 | 209.099 | 10.89 |
| 0.4333 | 945.49 | 1431.5 | 208.645 | 10.76 |
| 0.5840 | 949.35 | 1440.1 | 207.797 | 10.55 |
| 0.7337 | 952.91 | 1447.9 | 207.021 | 10.36 |
| 1.0917 | 960.49 | 1466.0 | 205.389 | 9.94 |
| **T = 323.15 K** | | | | |
| 0.0752 | 925.74 | 1367.1 | 213.106 | 12.32 |
| 0.1519 | 927.62 | 1374.5 | 212.670 | 12.13 |
| 0.2181 | 929.34 | 1377.6 | 212.275 | 12.03 |
| 0.2916 | 931.31 | 1381.4 | 211.825 | 11.91 |
| 0.3686 | 934.20 | 1389.1 | 211.166 | 11.71 |

| | | | | |
|---|---|---|---|---|
| 0.4333 | 936.28 | 1394.5 | 210.696 | 11.56 |
| 0.5840 | 940.28 | 1403.5 | 209.800 | 11.32 |
| 0.7337 | 943.94 | 1411.5 | 208.987 | 11.11 |
| 1.0917 | 951.76 | 1430.3 | 207.272 | 10.64 |
| | | **T = 343.15 K** | | |
| 0.0752 | 906.43 | 1291.8 | 217.642 | 14.38 |
| 0.1519 | 908.49 | 1298.6 | 217.145 | 14.17 |
| 0.2181 | 910.37 | 1302.9 | 216.695 | 14.01 |
| 0.2916 | 912.51 | 1308.2 | 216.185 | 13.83 |
| 0.3686 | 915.54 | 1315.4 | 215.466 | 13.59 |
| 0.4333 | 917.75 | 1320.1 | 214.947 | 13.43 |
| 0.5840 | 922.02 | 1329.9 | 213.952 | 13.11 |
| 0.7337 | 925.90 | 1339.7 | 213.056 | 12.81 |
| 1.0917 | 934.24 | 1360.4 | 211.156 | 12.20 |

Standard uncertainties $u$ is $u(\rho)$ = 0.94 kg·m$^{-3}$, $u(u)$ = 2.9m·s$^{-1}$; $u(m)$ = 0.0007 mol·kg$^{-1}$; $u(T)$ = 0.001 K, $u(p)$ = 0.01 MPa, $u(V_\phi)$ = 0.009 cm$^3$·mol$^{-1}$ and $u(K_\phi)$ = 0.08 x10$^{-5}$ cm$^3$.mol$^{-1}$.Pa$^{-1}$ (0.68 level of confidence).

**Table S2:** Total energies in Hartrees and kcal mol$^{-1}$ used to calculate $E_{inter}$

| Structure | Gas phase | | DMF | |
|---|---|---|---|---|
| | **Hartrees** | **kcal mol$^{-1}$** | **Hartrees** | **kcal mol$^{-1}$** |
| [Bmim] | -423,284507 | -265614,838 | -423,377581 | -265673,4182 |
| [Bmpym] | -409,644904 | -257055,864 | -409,736810 | -257113,5359 |
| $^{-}$SCN | -491,168763 | -308212,819 | -491,255428 | -308267,2024 |
| $^{-}$Cl | -460,303727 | -288844,731 | -460,408728 | -288910,6205 |
| DMF | -248,587511 | -155990,90 | -248,599407 | -155998,3653 |
| [Bmim][SCN] | -914,593637 | -573915,739 | -914,641218 | -573945,5961 |
| [Bmim][Cl] | -883,737494 | -554553,231 | -883,792852 | -554587,9688 |
| [Bmpym][Cl] | -870,094490 | -545992,123 | -870,150602 | -546027,3341 |
| [Bmim][SCN]-DMF | -1163,20515 | -729921,702 | -1163,25032 | -729950,0419 |
| [Bmim][Cl]-DMF | -1132,349038 | -710559,212 | -1132,400161 | -710591,2926 |
| [Bmpym][Cl]-DMF | -701968,0539 | -702000,344 | -1118,758875 | -702031,2629 |

### NCI and AIM analysis of [Bmim][Cl] + DMF in gas phase

In the [Bmim][Cl]-DMF complex (gas phase) the ionic liquid interacts with the DMF molecule through four specific interactions (Fig. S1a): the first two interactions are where the hydrogen atoms (H34 and H37) of DMF interact with the chloride anion (H34-Cl and H37-Cl); the second two interactions are where two hydrogen atoms (H14 and H20) from the cation of the ionic liquid interact with O38 of DMF via H14-O34 and H20-O38. In the molecular graph (Fig. S1b), the bond critical points (BCPs) of the [Bmim][Cl]-DMF complex are highlighted.

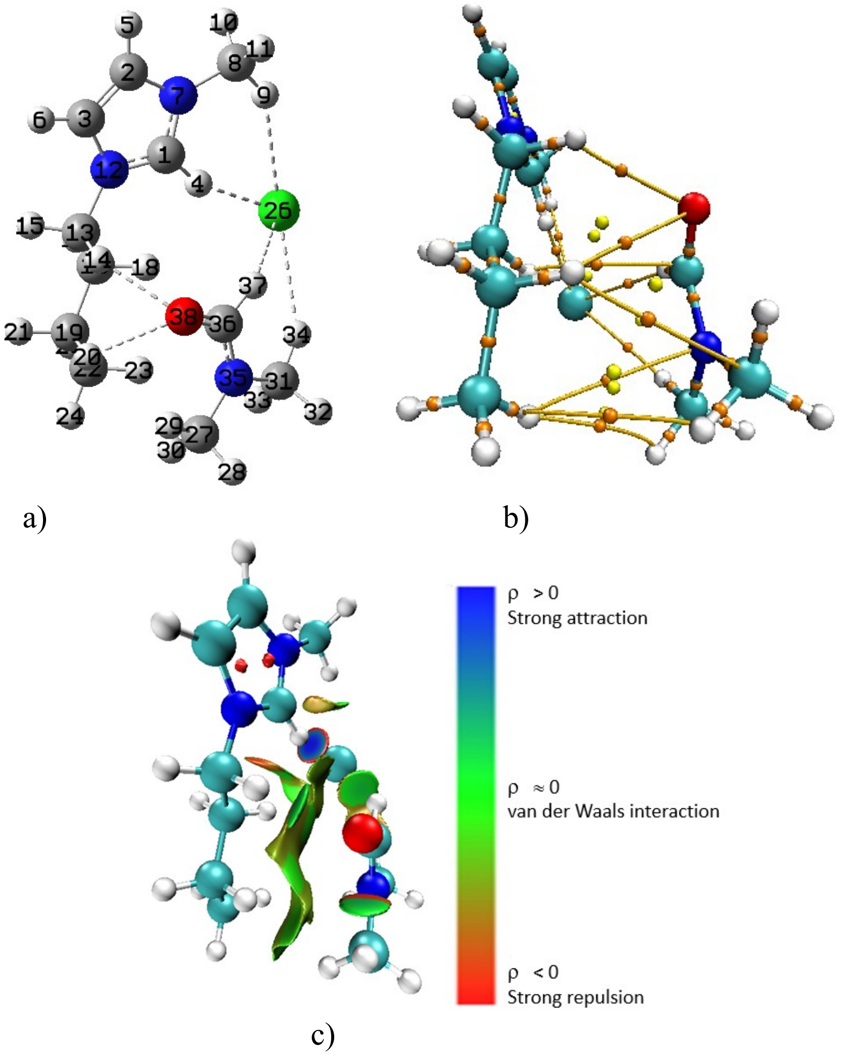


**Fig. S1.** a) Optimized structure [Bmim][Cl]-DMF in the gas phase b) Molecular graph with BCPs and c) 3D NCI plot.

The green isosurface observed between O38-H14, Cl-H37, Cl-H20 as well as Cl-H34, confirm the presence of non-covalent interactions. From the calculated values of ρ(r) and $\nabla^2\rho(r)$ (Table S3), the ρ(r) value is highest for the H14-O38 interaction which indicates that, among the four interactions, this interaction contributes the most to the interaction between the IL and DMF. The NCI plot (Fig. S1c) of [Bmim][Cl]-DMF also reveals a strong non-covalent interaction between the anion of the ionic liquid (Cl26) and H4 of the cation. This is evidenced by the blue isosurface (blue disk) in the 3D NCI plot and the highest electron density value of 0.0327 a.u. at the Cl26-H34 bond critical point (BCP).

**Table S3:** Bond distances and AIM analysis at selected BCPs for [Bmim][Cl]-DMF in gas phase

| BCP | Distance (Å) | ρ(r) (a.u.) | $\nabla^2\rho(r)$ (a.u.) |
|---|---|---|---|
| H34-Cl | 2.72 | 0.0098 | 0.0301 |
| H37-Cl | 2.68 | 0.0112 | 0.0334 |
| H14-O38 | 2.24 | 0.0147 | 0.0538 |
| H20-O38 | 2.79 | 0.0057 | 0.0184 |

**NCI and AIM analysis of [Bmim][Cl] + DMF in solvent**

The next part of the calculation was to add the IL complex in DMF solvent. As observed (Fig. S2a and b) there are three possible interactions when the complex is in the solvent environment: H14-O38; H20-N35 and H37-Cl26. Additionally, in solvent, the distance between H34 and Cl26 increases to 3.77 Å as compared to its gas phase value of 2.72 Å (Table S3). The distance between H20 and O38 increases to 2.90 Å in solvent whereas in the gas phase the value was 2.79 Å. The absence of a BCP between H20-O38, and H34-Cl26, in the molecular graph (Fig. S2b) confirm that the binding from the H20-O38 and H34-Cl26 interactions are negligible in this solvent. However, a new interaction between H20-N35 was observed in solvent, which is not present in the gas phase. This suggests that the solvent influences the how the molecule orients itself in solution.

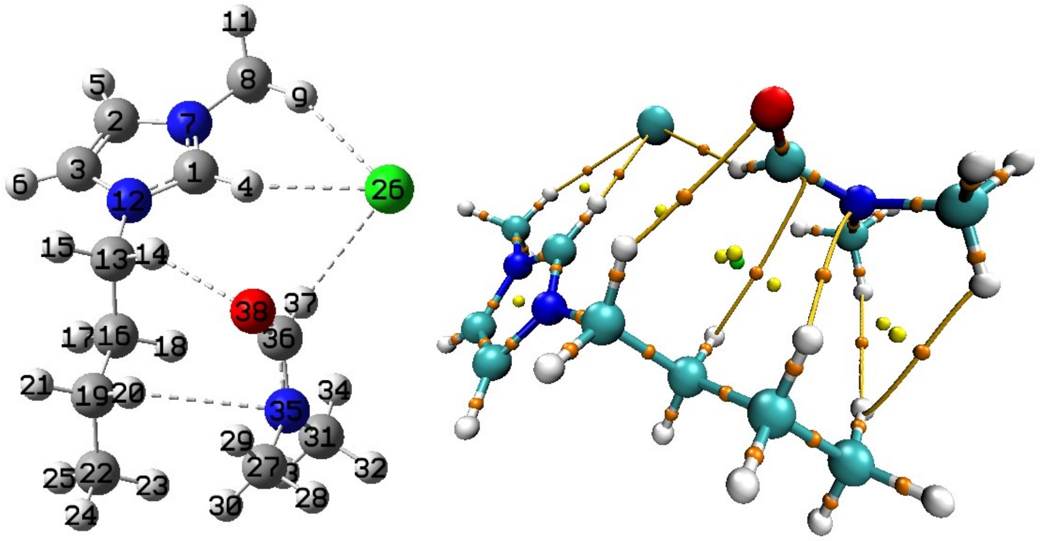


a) b)

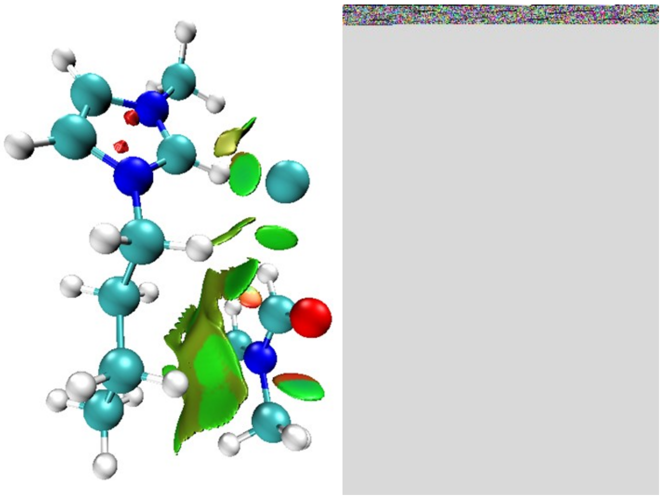

c)

**Fig. S2.** a) Optimized [Bmim][Cl]-DMF in DMF solvent b) Molecular graph with BCPs and c) 3D NCI plot.

All the $\nabla^2\rho(r)$ are positive (Table S4), indicating the interactions are non-covalent. The $\rho(r)$ values calculated at the corresponding BCPs range from 0.0055 to 0.0079 au, suggesting that these interactions are weak van der Waals type in nature. In the gas phase, a strong ion-dipole interaction was observed between the chloride anion (Cl26) and H4 of the cation of the ionic liquid. However, this interaction (H4-Cl26) weakened in solvent, as indicated by the light blue isosurface between these atoms in the NCI plot (Fig. S2c).

**Table S4:** Bond distances and AIM analysis at selected BCPs for [Bmim][Cl]-DMF in DMF solvent

| BCP | Distance (Å) | $\rho(r)$ (a.u.) | $\nabla^2\rho(r)$ (a.u.) |
|---|---|---|---|
| Cl26---H4 | 2.59 | 0.0121 | 0.0388 |
| Cl26---H9 | 2.83 | 0.0082 | 0.0238 |
| H37---Cl26 | 2.86 | 0.0078 | 0.0218 |
| H14---O38 | 2.57 | 0.0079 | 0.0248 |
| H20----N35 | 2.89 | 0.0055 | 0.0182 |

**NCI and AIM analysis of [Bmim][SCN] + DMF in gas phase**

Next, we examined the interactions for the [Bmim][SCN]-DMF complex. In the gas phase, there are four bond critical points (BCPs) in the molecular graph (Fig. S3a and b) which are N28-H38, N28-H32, O39-H9 and O39-C2. This suggests that four distinct interactions exist between pairs of atoms in the system. The values of $\nabla^2\rho(r)$ and H(r) for all the BCPs calculated

are positive, suggesting that these interactions are weak in the IL-DMF complex. As already mentioned, NCI plots assist in visualizing nature of interactions with the use of colour to differentiate the interactions. Red denotes steric clashes, green shows weak interactions, and blue indicates strong interactions. The green isosurfaces (Fig.S3c) between the interacting atoms in the [Bmim][SCN]-DMF complex confirms the presence of weak to medium hydrogen bonding. However, the ρ(r) value for O39-H9 for the DMF molecule is largest (0.0133 a.u.) (Table S5), suggesting that this is the strongest interaction in this IL-DMF interaction.

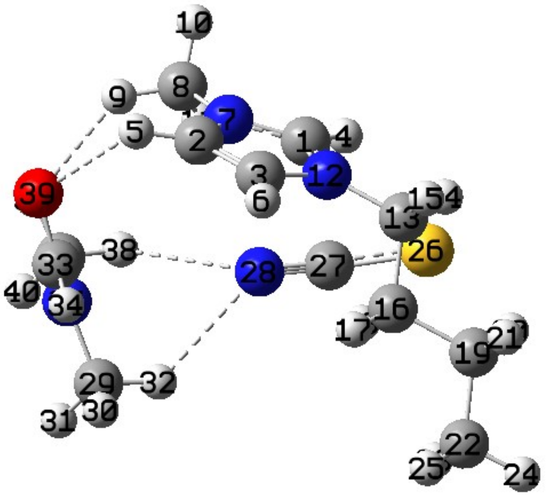


a)

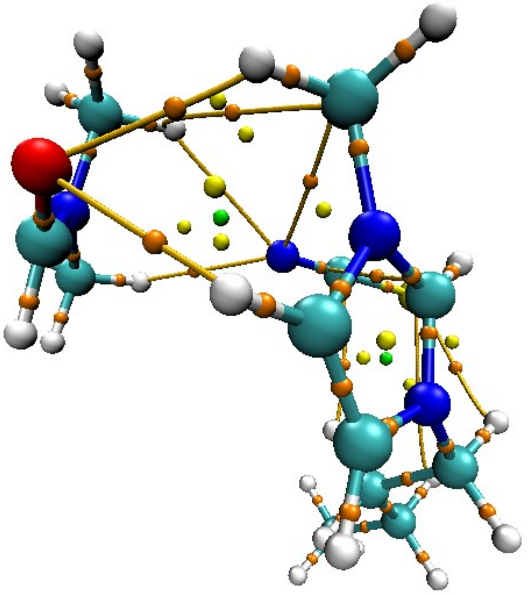

b)

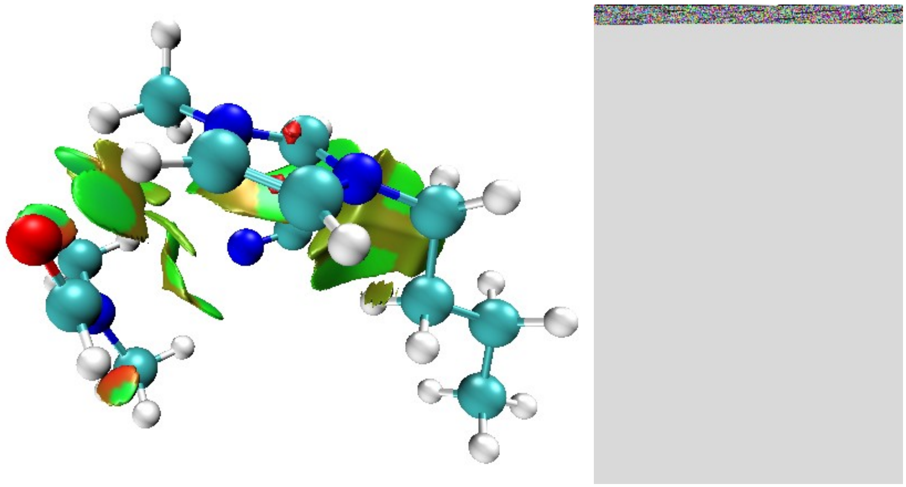

c)

**Fig. S3.** a) Optimized structure of [Bmim][SCN]-DMF in the gas phase b) Molecular graph with BCPs and c) 3D NCI plot.

**Table S5:** Bond distances and AIM analysis at selected BCPs for [Bmim][SCN]-DMF in gas phase

| BCP | Distance (Å) | ρ(r) (a.u.) | $\nabla^2\rho(r)$ (a.u.) | H | LOL |
|---|---|---|---|---|---|
| N28---H38 | 2.55 | 0.00944 | 0.0304 | 0.0012 | 0.1601 |
| N28---H32 | 2.48 | 0.0105 | 0.0344 | 0.0014 | 0.1677 |
| O39---H9 | 2.30 | 0.0133 | 0.0465 | 0.0016 | 0.1757 |
| O39---H5 | 2.41 | 0.0116 | 0.0416 | 0.0016 | 0.1623 |

**NCI and AIM analysis of [Bmim][SCN] + DMF in solvent**

When the complex was modelled in DMF solvent, the O39-H5 and N28-H38 interactions were not observed in DMF solvent. The bond distance between N28-H38 increased from 2.55 Å in the gas phase to 3.80 Å (Table S6) in the DMF solvent, which is further proof of the weakened bond in solvent. Similarly, the bond distance between O39-H9 also increased from 2.41 Å in the gas phase to 3.44 Å in DMF, indicating a weak interaction (Fig. S4a and b). Since the bond distances of these interactions are significantly elongated, the bond critical points (BCPs) between these atoms in the molecular graph, do not appear. The bond distance for N28-H32 and O39-H9 also increased in solvent compared to the gas-phase values. The N28-H32 interaction increased from 2.48 Å in the gas phase to 2.76 Å in solvent, while the O39-H9 interaction increased from 2.30 Å (gas) to 2.54 Å (solvent). The electron density ρ(r) at the bond critical point for N28-H32 and O39-H9 are 0.0061 a.u. and 0.0083 a.u., respectively in DMF which highlights the reduced interacting strength in solvent.

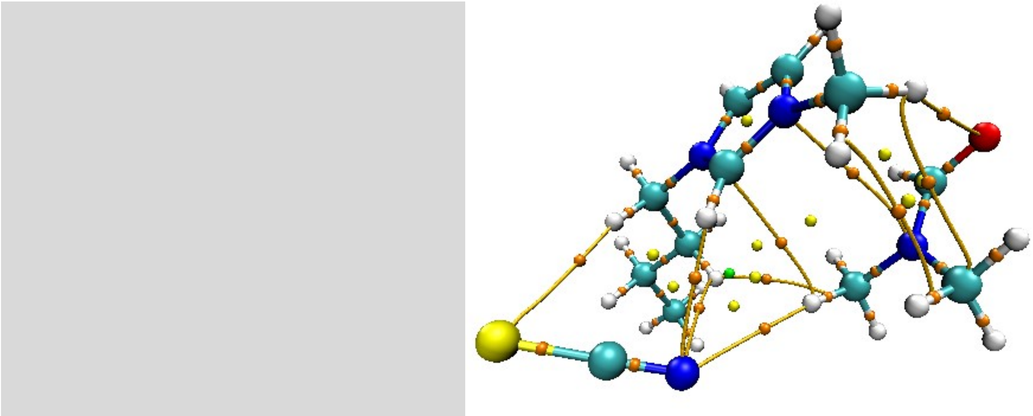

a) b)

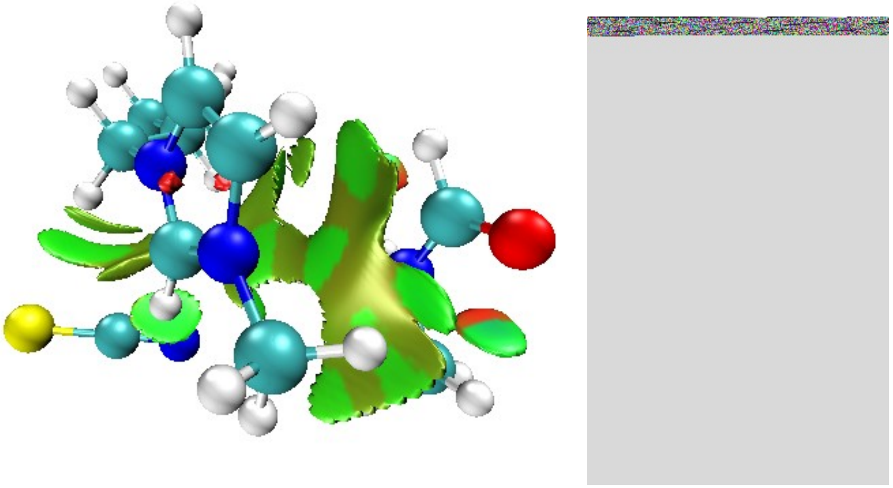

c)

**Fig. S4.** a) Optimized structure of [Bmim][SCN]-DMF in DMF solvent b) Molecular graph with BCPs and c) 3D NCI plot.

**Table S6:** Bond distances and AIM analysis at selected BCPs for [Bmim][SCN]-DMF in DMF solvent

| BCP | Distance (Å) | ρ(r) (a.u.) | $\nabla^2\rho(r)$(a.u.) |
|---|---|---|---|
| N28---H32 | 2.76 | 0.0061 | 0.0175 |
| O39---H9 | 2.54 | 0.0083 | 0.0265 |
| N7---N35 | 3.37 | 0.0058 | 0.0179 |

**NCI and AIM analysis of [Bmpym][Cl] + DMF in gas phase**

For the Bmpym][Cl]-DMF complex in the gas phase, five non-covalent interactions between DMF and the ionic liquid was observed (Fig. S5a and b). The interactions were between the chloride ion from the ionic liquid with H35 and H37 of the DMF molecule (Fig. S5c). Additionally, the O43 atom of the DMF molecule interacts with H7, H16 and H22 in the cation of the ionic liquid. The calculated $\nabla^2\rho(r)$ values at these BCPs are positive, indicating that these interactions are non-covalent (Table S7). The $\nabla^2\rho(r)$ values show that the O43-H16 interaction is the strongest and should contribute most to the binding of DMF molecule to the ionic liquid.

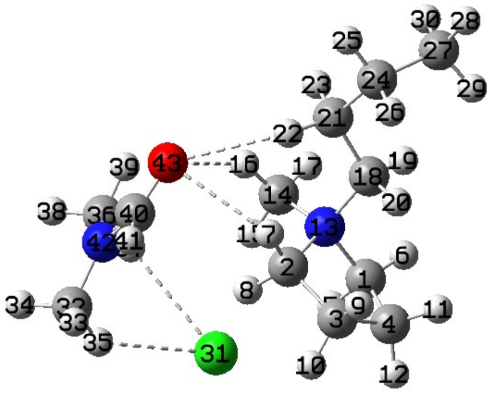

a)

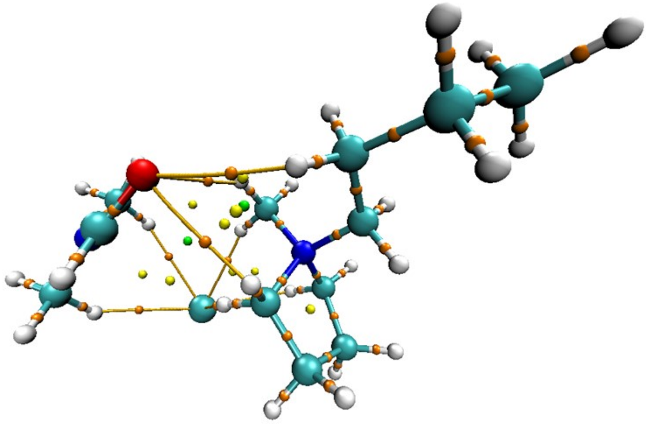

b)

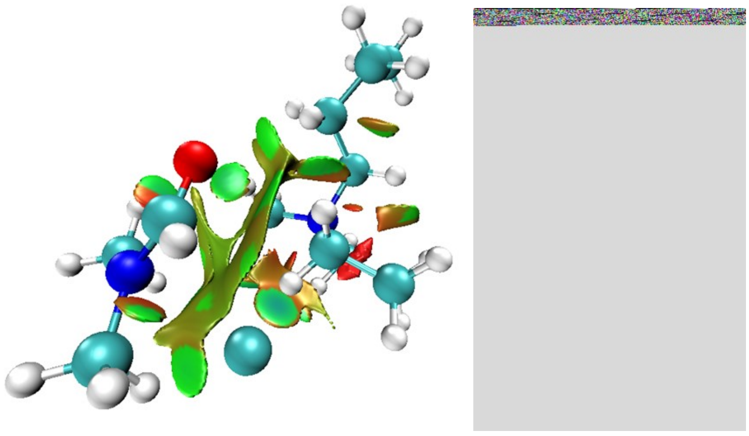

c)

**Fig. S5.** a) Optimized structure of [Bmpym][Cl]-DMF in the gas phase b) Molecular graph with BCPs and c) 3D NCI plot.

**Table S7:** Bond distances and AIM analysis at selected BCPs for [Bmpym][Cl]-DMF in gas phase.

| BCP | Distance (Å) | ρ(r) | $\nabla^2\rho(r)$ |
|---|---|---|---|
| H35---Cl31 | 2.74 | 0.0097 | 0.0291 |
| H37---Cl31 | 2.78 | 0.0094 | 0.0274 |
| O43---H7 | 2.59 | 0.0088 | 0.0289 |
| O43---H16 | 2.32 | 0.0129 | 0.0439 |
| O43---H22 | 2.58 | 0.0075 | 0.025 |

**NCI and AIM analysis of [Bmpym][Cl] + DMF in solvent**

The molecular graph of the [Bmpym][Cl]-DMF complex optimized in DMF solvent using the B3LYP-D3 method does not show the interactions: H35-Cl31, O43-H7, and O43-H22. The absence of this interaction can be attributed to the increased bond length between these interacting atoms in the complex. For instance, the O43-H22 distance increased significantly from its gas-phase value of 2.58 Å to 3.31 Å in DMF solvent. The H35-Cl31 bond length increased to 4.08 Å from its gas-phase value of 2.74 Å. However, QTAIM analysis identifies H37-Cl31 and O43-H16 interactions with electron densities (ρ(r)) of 0.0074 and 0.0077 au, respectively. These interactions contribute to binding DMF to the ionic liquid in the DMF solvent. A comparison showed the reduced ρ(r) values, compared to those in the gas phase, indicate that the interactions are weaker and are primarily of van der Waals type in nature.

**XYZ Coordinates for the gas phase optimised structures at B3LYP-D3/6-31++G(d,p)**

**[Bmim][SCN]-DMF**

E(B3LYP-D3) = -1163,205153 Hartree

| | | | |
|---|---|---|---|
| C | -1.03459400 | -1.27989300 | -0.66481700 |
| C | -0.33033200 | 0.57450900 | -1.63577700 |
| C | -0.00215900 | 0.63824500 | -0.31677200 |
| H | -1.51650000 | -2.22744000 | -0.47767200 |
| H | -0.22489800 | 1.28603200 | -2.43672700 |
| H | 0.49104100 | 1.40732300 | 0.25262500 |
| N | -0.97236400 | -0.63471700 | -1.83123200 |
| C | -1.61334700 | -1.09304400 | -3.06711500 |
| H | -1.74730100 | -0.23071800 | -3.71514100 |
| H | -0.99578900 | -1.84931700 | -3.55280900 |
| H | -2.59058700 | -1.49742500 | -2.80673300 |
| N | -0.44564900 | -0.53512500 | 0.27185000 |
| C | -0.48941200 | -0.82274200 | 1.71673900 |
| H | -0.74440600 | -1.87779300 | 1.81904100 |
| H | 0.51396100 | -0.66429000 | 2.12008600 |
| C | -1.53683600 | 0.03811400 | 2.42281100 |
| H | -1.24383300 | 1.09437200 | 2.37173200 |
| H | -2.48256600 | -0.06160800 | 1.88432500 |
| C | -1.72635000 | -0.38927000 | 3.87958700 |
| H | -2.02018200 | -1.44414000 | 3.89694900 |
| H | -0.77348400 | -0.31490700 | 4.42012100 |
| C | -2.79281900 | 0.44592800 | 4.59002900 |
| H | -3.76014500 | 0.34565000 | 4.08979200 |
| H | -2.92128600 | 0.12673800 | 5.62770900 |
| H | -2.52941600 | 1.50886200 | 4.59994800 |
| S | -3.56309400 | -2.89942800 | 1.21868600 |

| | | | |
|---|---|---|---|
| C | -3.86421600 | -1.65342100 | 0.17207500 |
| N | -3.98481600 | -0.76590100 | -0.59239100 |
| C | -5.14990200 | 2.22297500 | -1.39917700 |
| H | -4.66624500 | 3.00591600 | -0.81291600 |
| H | -6.12286300 | 2.59316600 | -1.73603900 |
| H | -5.28549500 | 1.34051600 | -0.77077800 |
| C | -3.06567700 | 2.34090900 | -2.67219000 |
| H | -2.81136400 | 3.09066100 | -1.90297400 |
| N | -4.32131000 | 1.88017800 | -2.54714900 |
| C | -4.83378200 | 0.83732300 | -3.42550500 |
| H | -4.16056300 | 0.72133100 | -4.27232500 |
| H | -4.90772900 | -0.10024800 | -2.86983200 |
| O | -2.24550800 | 2.00859900 | -3.52793900 |
| H | -5.82422300 | 1.12096100 | -3.79250100 |

**[Bmim][Cl]-DMF**

E(B3LYP) = -1132,34903 Hartree

| | | | |
|---|---|---|---|
| C | -0.40354300 | -1.90475900 | -0.73466200 |
| C | -0.75069700 | -4.09138600 | -0.78121600 |
| C | -0.95922300 | -3.65802000 | 0.49233500 |
| H | -0.15902400 | -0.89723400 | -1.10237300 |
| H | -0.81248700 | -5.07652300 | -1.21235800 |
| H | -1.24770100 | -4.19298600 | 1.38140800 |
| N | -0.40456500 | -2.98049000 | -1.52891300 |
| C | -0.07995100 | -2.94128200 | -2.95970400 |
| H | 0.15098900 | -1.90829600 | -3.22623000 |
| H | 0.78352600 | -3.57802200 | -3.15361600 |
| H | -0.93762100 | -3.29069100 | -3.53492100 |
| N | -0.73187300 | -2.29289900 | 0.50101200 |
| C | -0.92940600 | -1.38286000 | 1.65118200 |
| H | -1.95631500 | -1.01870300 | 1.60176900 |
| H | -0.80459500 | -1.98864300 | 2.55174900 |
| C | 0.04478800 | -0.20834900 | 1.63849700 |
| H | 1.07426100 | -0.57578800 | 1.72129700 |
| H | -0.01569800 | 0.32047900 | 0.68314800 |
| C | -0.26641900 | 0.77860300 | 2.76732200 |
| H | -1.31624800 | 1.07869000 | 2.69032100 |
| H | -0.15484700 | 0.28357300 | 3.74045800 |
| C | 0.62951500 | 2.01688800 | 2.70844100 |
| H | 0.51164700 | 2.53706900 | 1.75361800 |
| H | 0.38833500 | 2.72178300 | 3.50899000 |
| H | 1.68675400 | 1.75232500 | 2.80671400 |
| Cl | 0.37230500 | 0.66527800 | -2.42538800 |
| C | -2.65869400 | 3.32079500 | 1.30800100 |
| H | -3.25753900 | 4.20021600 | 1.04925200 |
| H | -3.28562000 | 2.60371400 | 1.83417300 |

| | | | |
|---|---|---|---|
| H | -1.84401400 | 3.64134600 | 1.96596300 |
| C | -1.32356600 | 3.52937400 | -0.78081700 |
| H | -1.92922900 | 4.33024700 | -1.21928300 |
| H | -0.50346500 | 3.98673400 | -0.21804200 |
| H | -0.88853800 | 2.91758400 | -1.57149900 |
| N | -2.13061900 | 2.70073500 | 0.10592500 |
| C | -2.39094200 | 1.40534200 | -0.18964600 |
| H | -1.94187600 | 1.09141200 | -1.14207400 |
| O | -3.02989800 | 0.62982000 | 0.52226300 |

**[Bmpym][Cl]-DMF**

E(B3LYP) = -1118,709602 Hartree

| | | | |
|---|---|---|---|
| C | -0.82172000 | -2.98577400 | -0.60909000 |
| C | -1.44134900 | -0.77025500 | -0.04996400 |
| C | -1.60462100 | -1.58976100 | 1.23555600 |
| C | -1.12657200 | -3.02596100 | 0.88769200 |
| H | -1.72872300 | -3.17284800 | -1.18647600 |
| H | -0.01497600 | -3.63905800 | -0.94253000 |
| H | -1.08097500 | 0.24513900 | 0.08109500 |
| H | -2.36988400 | -0.77158200 | -0.62174800 |
| H | -1.02741400 | -1.17142500 | 2.06331900 |
| H | -2.65472200 | -1.58078600 | 1.52769600 |
| H | -0.24692500 | -3.30375600 | 1.47331200 |
| H | -1.89798400 | -3.77086900 | 1.08253500 |
| N | -0.43964700 | -1.54168500 | -0.90258700 |
| C | -0.60077000 | -1.24251100 | -2.36446400 |
| H | -1.60171700 | -1.55919200 | -2.65604200 |
| H | -0.49025700 | -0.17335500 | -2.51918500 |
| H | 0.16013800 | -1.79887200 | -2.91347900 |
| C | 0.98121200 | -1.26810200 | -0.45101500 |
| H | 1.59514800 | -2.05879700 | -0.88540700 |
| H | 0.99523500 | -1.40082800 | 0.63203500 |
| C | 1.52209700 | 0.11187500 | -0.82908400 |
| H | 0.75735400 | 0.87925100 | -0.68964400 |
| H | 1.77154400 | 0.13088200 | -1.89410500 |
| C | 2.77028100 | 0.47456500 | -0.01131800 |
| H | 3.06841700 | 1.49131800 | -0.28550000 |
| H | 2.50915800 | 0.51481600 | 1.05363800 |
| C | 3.95511600 | -0.47414900 | -0.21839000 |
| H | 4.84026300 | -0.12233000 | 0.31751700 |
| H | 3.74335300 | -1.48588500 | 0.14111500 |
| H | 4.21973200 | -0.54888800 | -1.27824700 |
| Cl | -3.95628200 | -2.12962600 | -1.86446400 |
| C | -4.84989900 | 1.39354400 | -2.07218000 |
| H | -4.97025200 | 1.86982400 | -1.09780000 |
| H | -5.49579100 | 1.90502600 | -2.79229300 |

| | | | |
|---|---|---|---|
| H | -5.13959200 | 0.34231500 | -1.99503400 |
| C | -3.14419400 | 0.88402800 | -3.79069600 |
| H | -3.37300000 | -0.18449900 | -3.76067900 |
| H | -3.74751600 | 1.36043000 | -4.56921200 |
| H | -2.09075000 | 1.04239900 | -4.01137200 |
| C | -2.49429000 | 1.93217900 | -1.67830600 |
| H | -2.90112100 | 2.33970200 | -0.73604300 |
| N | -3.45784700 | 1.48312400 | -2.49926000 |
| O | -1.28051000 | 1.91472000 | -1.88709700 |
